# Reliability-Guided Dynamic Fusion for Few-Shot Writer-Specific Stroke-Width Estimation in Offline Handwriting Biometric Sensing †

**DOI:** 10.3390/s26196355

**Published:** 2026-10-08

**Authors:** Jihan Liu

**Affiliations:** College of Artificial Intelligence, Tianjin University of Science and Technology, Tianjin 300457, China; 24106204@mail.tust.edu.cn

**Keywords:** biometric sensing, handwriting biometrics, stroke-width estimation, few-shot adaptation, confidence-guided fusion, writer-disjoint evaluation, verification robustness

## Abstract

**Highlights:**

**What are the main findings?**

- Deterministic confidence-guided dynamic fusion (primary operating point) consistently outperforms validation-selected fixed-β and model-only baselines on the pseudo-label protocol under a 73-writer writer-disjoint setting.
- Three-layer evidence structure: (i) primary protocol claims; (ii) manual-click audit of circularity/scale (not a fair fusion ranking); (iii) learned gates as an optional enhancement that can lower pseudo-label MAE but need fusion-time training and do not transfer under a generator swap.

**What are the implications of the main findings?**

- It supports training-free, inspectable few-shot writer-specific stroke-width estimation for offline handwriting biometric sensing with limited enrollment samples.
- It provides a reproducible rendering stack for evaluating personalization fidelity and verification robustness in handwriting biometrics.

**Abstract:**

Offline handwriting functions as a practical biometric sensing modality for identity verification and controlled stress testing of recognition pipelines, yet recovering writer-specific stroke appearance from only a few enrollment samples remains difficult. In trajectory-based rendering used to probe such sensing systems, stroke width is often fixed or only weakly adapted, so the sensed thickness cue fails to reflect individual writing habits. The key obstacle is an operational reliability imbalance: a learned width estimator and handcrafted geometric priors disagree in trustworthiness across writers and even along a single stroke, which makes a constant fusion rule brittle for few-shot personalization. We propose a confidence-guided dynamic fusion framework whose primary method is a deterministic confidence-to-β map (not a learned gate): point-wise pseudo-label confidence from reference images sets per-point blending weights between learned and rule-based widths, with “reliability” denoting this operational blending prior. On 73 HWDB writers under a strict writer-disjoint protocol with 240 adaptation runs, dynamic fusion reduces writer-level variance and cross-seed instability relative to fixed fusion while retaining competitive point-wise accuracy. A security-oriented sensing analysis further contrasts detector responses under generic versus writer-adapted rendering, showing that the framework can serve as a controllable probe of verification sensitivity. The method plugs into existing trajectory generators and supports reproducible evaluation of personalization fidelity and biometric sensing robustness.

## 1. Introduction

Trajectory-based handwriting synthesis has made significant progress in reproducing pen movement patterns and stroke sequences [1,2,3,4]. However, generating handwriting that faithfully preserves writer-specific visual characteristics remains challenging, particularly when only a limited number of samples are available for adaptation. Unlike trajectory generation, appearance-related attributes such as stroke width are highly dependent on individual writing habits and exhibit substantial variations among writers [5,6,7]. Modeling writer-specific stroke-width characteristics under few-shot scenarios remains an open problem.

Existing handwriting synthesis approaches mainly focus on trajectory modeling and sequence generation, while stroke appearance adaptation has received relatively less attention. A small body of work does target stroke thickness explicitly—for example, quill-inspired width modeling in calligraphy-oriented generation [8]—but such appearance-centric studies remain far fewer than trajectory- or pixel-generation pipelines surveyed elsewhere [5]. Moreover, existing width-focused methods are often tailored to particular calligraphy styles or rendering setups and have not been widely integrated into general-purpose trajectory generators used for writer-specific few-shot synthesis [1,8]. Recent contour-aware trajectory reconstruction further underscores that stroke appearance beyond centerline geometry is still an open modeling gap [7]. A common strategy is to estimate stroke width using either learned predictors or manually designed priors derived from handwriting structures [9,10,11]. However, these two sources do not always provide consistent information. The reliability of a learned predictor may degrade when the available writer samples are insufficient, whereas handcrafted priors may fail to capture individual writing styles. More critically, the optimal balance between these sources varies across writers, making fixed fusion strategies—including constant-ratio blending used in our conference precursor [11] and fixed late-fusion weights common in multimodal analysis [12]—inherently suboptimal for few-shot personalization.

To address this issue, we introduce a confidence-guided dynamic fusion framework for few-shot writer-specific stroke-width adaptation. In this manuscript, “reliability” denotes an operational, confidence-guided prior for blending rather than a claim of branch Bayes-optimality: unstable confidence regions are pulled more toward the geometric rule, while high-agreement regions allow a larger weight on the learned branch. Instead of assigning a fixed fusion ratio between different stroke-width sources, the proposed framework estimates point-wise confidence and maps it deterministically to dynamically adjusted contributions of each source. The primary proposed method is this deterministic confidence-to-β mapping from multi-cue pseudo-label confidence heuristics, not a learned gating network; learned gates are reported later as an optional comparative enhancement. This design enables the model to adaptively select more reliable stroke-width information under different writer conditions. In addition, an efficient adaptation strategy is incorporated to reduce overfitting risks caused by extremely limited writer-specific samples. Compared with our ICICS 2026 conference precursor [11], this journal study adds writer-disjoint evaluation at scale, formal reliability modeling, parameter-efficient adaptation analysis, bootstrap confidence-interval quantification, imaging-robustness tests, and external diffusion baselines under a unified protocol.

We conduct experiments under a writer-disjoint protocol with 73 writers and multiple shot counts. The primary endpoints are fusion-stack stability metrics (writer-level standard deviation, cross-seed coefficient of variation, and worst-writer error), together with deployed-stack point-wise MAE after val-tuned fusion and post-processing. The results demonstrate that dynamic fusion provides more stable and more accurate deployed rendering than fixed fusion across these metrics. Furthermore, security-oriented evaluations with multiple handwriting verification systems are performed to quantify how generic versus adapted-fused rendering shifts detector responses under strict verification scenarios.

The main contributions of this work are summarized as follows:

1. We identify the **reliability imbalance** problem between learned stroke-width estimation and handcrafted stroke-width priors in few-shot writer-specific adaptation scenarios, interpreting reliability operationally as a confidence-guided blending prior.
2. We propose a confidence-guided dynamic fusion strategy that adaptively determines the contribution of different stroke-width sources according to a deterministic confidence-to-β map.
3. We establish a writer-disjoint evaluation protocol with 73 writers, including stability and security-oriented analyses to assess both personalization consistency and verification system sensitivity.

Our framework is designed as a reliability-aware combination mechanism for the deployed rendering stack rather than a replacement for generic predictors or an end-to-end generator. We explicitly distinguish between predictor-level accuracy and fused-pipeline stability, and position the latter as the primary contribution.

We model observable local stroke width rather than physical pen pressure, modify only the width module of a fixed trajectory generator, and report verification outcomes as *exploratory security-oriented analyses* rather than claims of real-world spoofing success.

### Related Work

**Handwriting synthesis.** Offline GAN and diffusion models [13,14,15,16] generate pixels directly but offer limited control over stroke order. Among them, One-DM and DiffBrush are particularly relevant Chinese handwriting synthesizers and serve as external baselines later in our evaluation [14,17]. Trajectory-based approaches [1,4,18] expose pen motion and enable modular rendering; recent open-set online generators further push unseen-writer/unseen-character settings [4], while contour-aware reconstruction emphasizes stroke appearance beyond centerlines [7]. Our work improves only the width module of a fixed trajectory pipeline.

**Stroke-width and pseudo-labels.** Classical pipelines estimate local radius via binarization and Euclidean distance transform [9,10]. Calligraphy-oriented generators further highlight stroke width as an appearance cue beyond trajectory geometry [8]. We treat pseudo-widths as noisy supervision and explicitly model reliability rather than assuming ground-truth.

**Few-shot adaptation.** Meta-learning and parameter-efficient fine-tuning (PEFT) [19,20,21] personalize models from few samples. We adapt a continuous width regressor with writer embeddings and parameter anchoring ($\lambda =10^{-4}$), targeting unseen characters after *k* reference images.

**Biometric forensics.** Detector outcomes depend strongly on architecture and features [22,23,24]. Controlled comparisons across CNN, residual, lightweight, and hybrid backbones further motivate reporting multiple verifiers rather than a single ASR figure [24]. We therefore report multiple verifiers and FAR operating points.

## 2. Reliability-Guided Dynamic Fusion Framework

### 2.1. Reliability Modeling Motivation

Different stroke-width sources provide complementary information but suffer from different failure modes. The learned predictor captures writer-specific patterns from data-driven training, benefiting from large-scale writer-independent prior knowledge. The handcrafted prior, on the other hand, provides structural regularity derived from handwriting geometry, ensuring smoothness and avoiding extreme deviations.

However, the reliability of these two sources is not constant across writers or stroke positions. For a writer whose style is well represented in the training data, the learned predictor may be highly reliable; for an unseen writer with unusual stroke habits, it may degrade. Similarly, the handcrafted prior performs well on regular strokes but fails at stroke junctions or endpoints where geometric assumptions break down. Crucially, the optimal balance between these sources varies from writer to writer, so a fixed fusion coefficient cannot accommodate this heterogeneity.

Therefore, instead of searching for a globally optimal fusion coefficient, we formulate the fusion process as a reliability-aware adaptive combination problem: the model should estimate the reliability of each source at each trajectory point and adjust the fusion weights accordingly. Again, “reliability” here is an operational label for the confidence-guided blending prior, not a necessary implication that high confidence entails branch superiority. This formulation treats reliability as a writer-dependent and spatially varying quantity, which is the central premise of our framework.

### 2.2. Framework Overview

**Figure 1:**
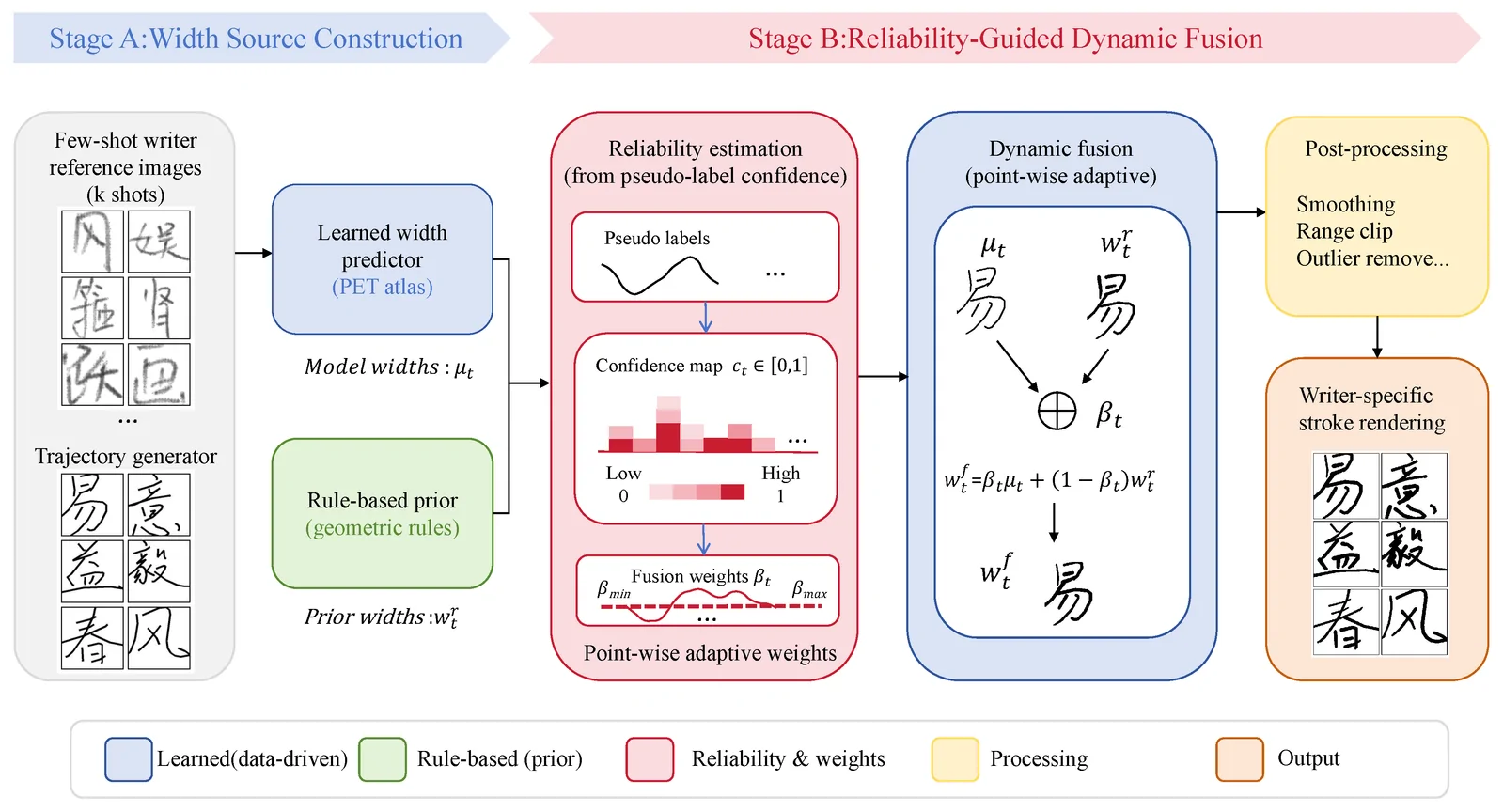
Overview of the proposed reliability-guided dynamic fusion framework for few-shot writer-specific stroke-width adaptation.

The overall framework aims to address the reliability imbalance problem in few-shot writer-specific stroke-width adaptation. As illustrated in Figure 1, the proposed framework consists of three sequential stages: initial stroke-width estimation, writer-specific adaptation, and reliability-guided dynamic fusion.

First, an initial stroke-width representation is obtained from handwriting trajectories through pseudo supervision. Then, a parameter-efficient adaptation strategy is employed to capture writer-specific characteristics from limited samples. Finally, instead of applying a fixed fusion ratio, the proposed dynamic fusion module estimates the reliability of different stroke-width sources and adaptively combines them to generate the final writer-aware stroke-width representation.

### 2.3. Stroke-Width Representation and Initial Estimation

**Figure 2:**
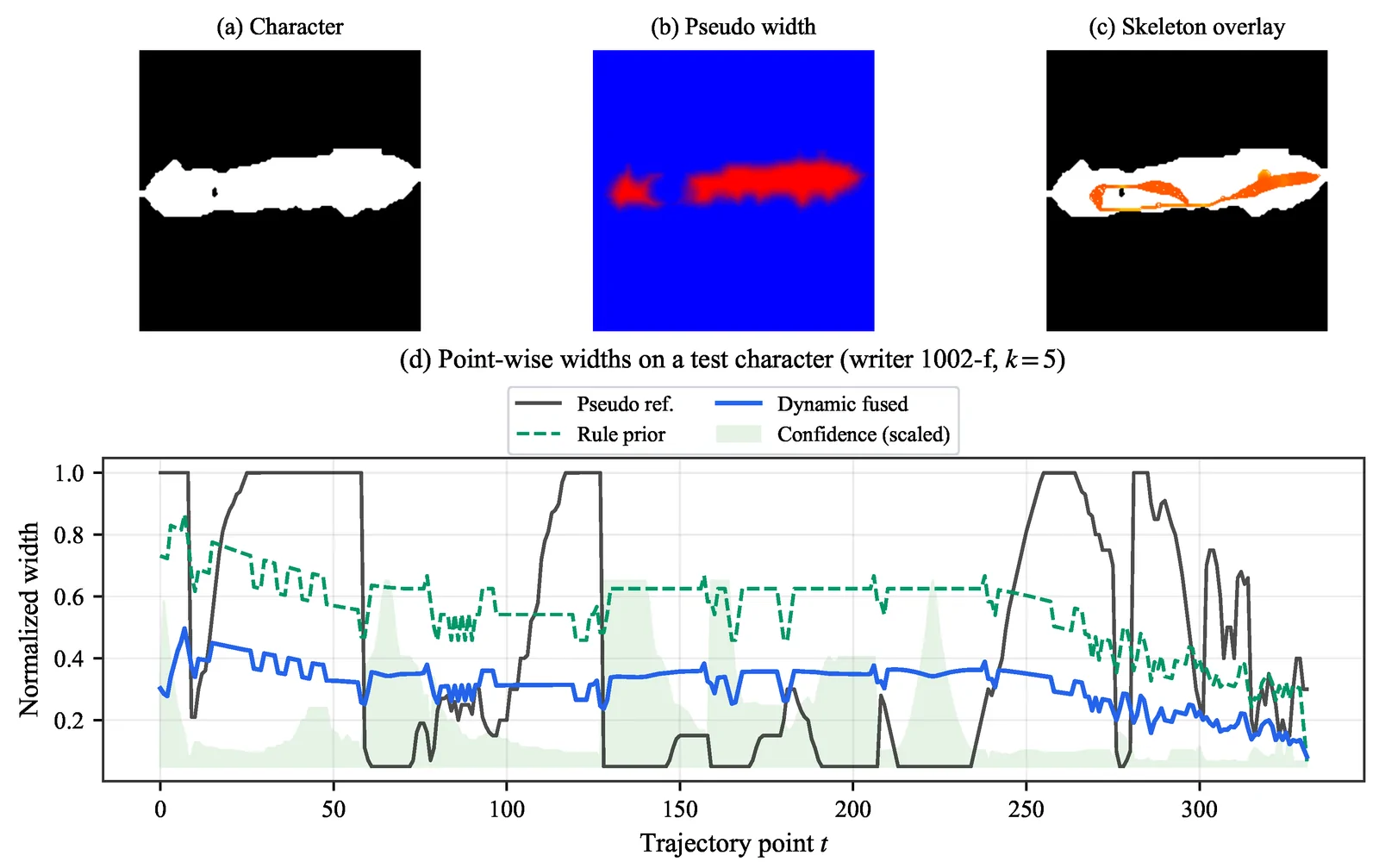
Stroke-width representation on a HWDB character. (**a**–**c**) Input character, pseudo-width map, and skeleton overlay. (**d**) Point-wise pseudo reference, rule prior, and dynamic fused widths along the trajectory; shaded region scales local confidence.

Stroke width provides important visual information for distinguishing individual writing styles [6,25]. However, directly obtaining pixel-level stroke-width annotations is expensive and impractical, especially in few-shot writer adaptation scenarios. Therefore, we construct pseudo stroke-width supervision from handwriting structures to provide an initial estimation of stroke-width characteristics. Figure 2 illustrates the object of study: pseudo-widths extracted along the pen trajectory, their spatial reliability, and how fused widths track reference thickness on a representative character.

For each reference image we compute pseudo-width ${\tilde{w}}_{t}$ and a confidence score $c_{t}\in \left[0,1\right]$ from multi-cue signals: label variance across binarization branches, edge symmetry [26], skeleton stability, and local contrast [9,10]. Low confidence points (typically bifurcations, stroke ends, and blurred regions) receive reduced loss weight:

(1) $$L_{\mathrm{pseudo}}=\frac{\sum_{t}c_{t}\cdot \rho \;\left(f_{\theta }\left(x_{t}\right)-{\tilde{w}}_{t}\right)}{\sum_{t}c_{t}+\varepsilon },$$

where ρ is a robust loss (Huber/Charbonnier). The obtained stroke-width representation serves as an initial prior rather than a final prediction, since its reliability may vary among different writers. Rulebased widths $w_{t}^{\mathrm{rule}}$ follow deterministic geometric rules on the same skeleton; learned widths ${\hat{w}}_{t}$ are regressed from trajectory features and writer context.

### 2.4. Few-Shot Writer Adaptation

Due to the limited number of writer-specific samples, directly optimizing all model parameters may introduce overfitting. Therefore, we adopt a parameter-efficient adaptation strategy to adjust writer-related representations while preserving the general knowledge learned from the base model [19,20,21]. A writer embedding $z_{s}$ from reference images modulates BiLSTM states via FiLM [27], $h_{t}^{\prime }=\gamma \left(z_{s}\right)⊙h_{t}+\beta \left(z_{s}\right)$, updating only adapters, FiLM layers, and embeddings (31.5% of parameters) with anchor regularization $\lambda ∥\theta -\theta_{0}{∥}^{2}$, $\lambda =10^{-4}$. Full fine-tuning (Conf FT) is retained as a baseline to evaluate whether parameter-efficient adaptation provides advantages under limited samples. The adapted predictor provides writer-specific stroke-width estimation, which serves as another information source for subsequent reliability-guided fusion.

### 2.5. Reliability-Guided Dynamic Fusion

Although both the initial stroke-width prior and writer-adapted prediction provide useful information, their reliability is not necessarily consistent across writers. A fixed fusion coefficient assumes that different sources have identical reliability distributions, which is unrealistic under few-shot scenarios. Therefore, we introduce a confidence-guided dynamic fusion strategy to estimate writer-dependent reliability and automatically adjust the fusion weights. The primary proposed method is a deterministic confidence-to-β mapping from multi-cue pseudo-label confidence heuristics, not a learned gating network.

After clipping and temporal smoothing, model widths $\mu_{t}$ are combined with rule prior $w_{t}^{\mathrm{rule}}$ through a point-wise coefficient $\beta_{t}\in \left[\beta_{\min},\beta_{\max}\right]$:

(2) $$w_{t}^{\mathrm{fused}}=\beta_{t}\;\mu_{t}+\left(1-\beta_{t}\right)\;w_{t}^{\mathrm{rule}},\;\beta_{t}=\beta_{\min}+\left(\beta_{\max}-\beta_{\min}\right)\;{\bar{c}}_{t},$$

where ${\bar{c}}_{t}$ is a smoothed confidence map along the stroke. Instead of manually selecting β, the proposed method determines $\beta_{t}$ according to the estimated reliability of each source. A larger $\beta_{t}$ indicates that the corresponding stroke-width source is considered more reliable for the current writer and stroke segment, whereas a smaller $\beta_{t}$ indicates lower reliability and greater reliance on the structural prior. **Fixed fusion** sets $\beta_{t}\equiv \beta_{0}$ everywhere and serves as the primary baseline representing reliability-agnostic blending. Hyperparameters $\left(\beta_{\min},\beta_{\max},\mathrm{smooth}\_\mathrm{kernel}\right)$ are selected on validation writers only; each test writer is evaluated once. Optional temporal smoothing uses kernel size 3 by default; cue-only ablations later show that smoothing has near-zero MAE effect and is presented as optional post-processing rather than an accuracy contribution.

**Figure 3:**
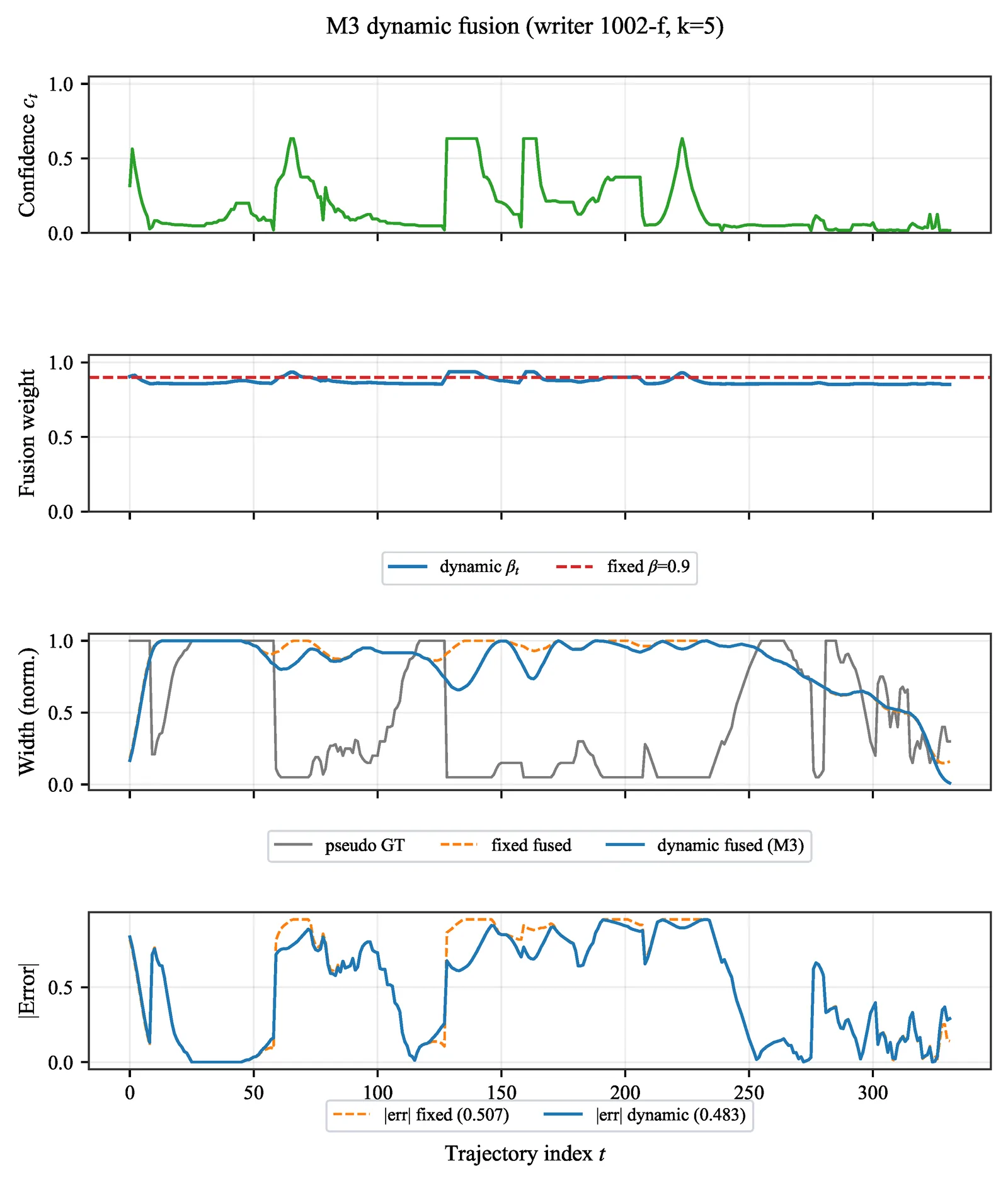
Reliability-guided dynamic fusion on a representative test character. (**a**) Pseudo-label confidence $c_{t}$. (**b**) Dynamic $\beta_{t}$ vs. an illustrative fixed reference β=0.7; fusion weights vary along the stroke instead of remaining constant. (**c**) Fused width vs. pseudo reference. (**d**) Point-wise absolute error (dynamic fusion reduces MAE from 0.507 to 0.483 on this example).

Figure 3 illustrates the mechanism on a representative character: confidence peaks at structurally stable stroke segments, $\beta_{t}$ varies accordingly, and dynamic fusion reduces point-wise error relative to an illustrative fixed reference (β=0.7 in the panel).

### 2.6. Discussion of the Framework

The proposed framework is not designed to replace large-scale handwriting generation models. Instead, it provides a lightweight and interpretable adaptation strategy for situations where writer-specific data are limited and different stroke-width sources exhibit inconsistent reliability. Reliability imbalance manifests both spatially (confidence is high on long stable segments but low near junctions and stroke terminals; see the later confidence-map and failure-audit figures) and across writers, where adaptation quality varies with support quality and individual stroke habits. Fixed fusion applies the same trade-off everywhere and therefore cannot track this heterogeneity, consistent with its fused-pipeline MAE of 0.303 vs. 0.292 for dynamic fusion at k=5 (reported in the later fused-pipeline MAE table). The framework therefore targets reliability-aware combination in the deployed rendering stack rather than replacing generic predictors or pursuing lowest average point-wise error in isolation.

## 3. Experimental Setup

### 3.1. Dataset and Evaluation Protocol

The experiments are conducted under a writer-disjoint evaluation protocol to simulate realistic unseen-writer adaptation scenarios. Specifically, writers appearing in the training stage are completely separated from those used for evaluation. This setting prevents information leakage and provides a more reliable assessment of few-shot writer-specific adaptation ability.

**Figure 4:**
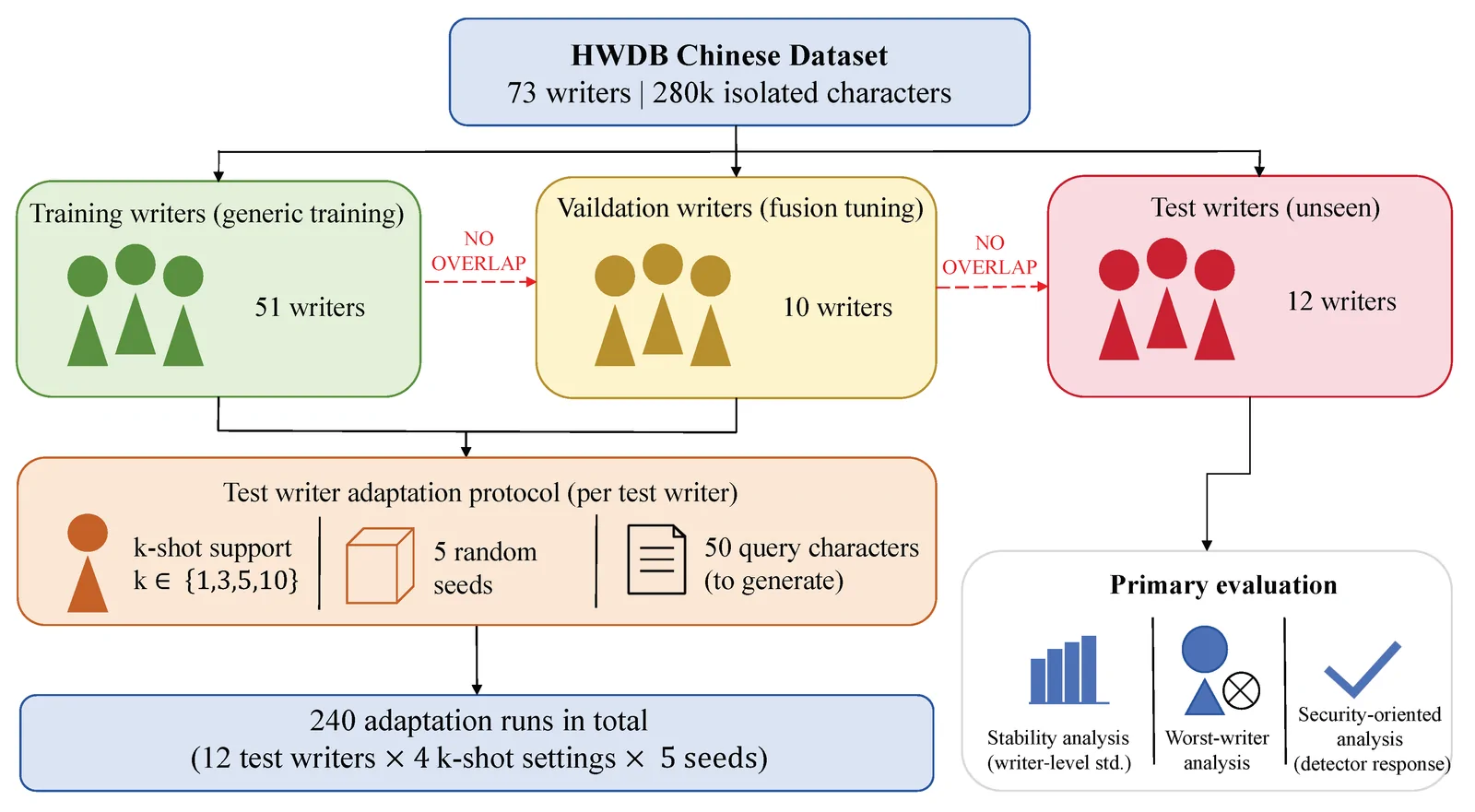
Writer-disjoint protocol: 73 HWDB writers split into train/validation/test with no overlap; each unseen test writer contributes 20 runs across *k* and seeds (240 runs total). A supplementary expanded HWDB1.1 check (180 writers; 28 test; k=5; seeds 0–1) is reported later in the expanded-split results table.

We use HWDB offline characters from 73 writers (∼280 k images) from the CASIA Chinese handwriting databases [28]. Writers are split 51/10/12 for train/validation/test with no overlap (writer_disjoint_v2.json). Training learns generic predictors; validation selects fusion hyperparameters; test reports final numbers once. For each *unseen* test writer we sample k∈{1,3,5,10} support images and five random seeds, yielding 240 adaptation runs (12×4×5). Support and query splits are within held-out writers with disjoint writer IDs from training; query characters are not used as PEFT support in the same run, so few-shot adaptation is not character memorization of the evaluation set. The SDT trajectory generator [1] produces cached trajectories shared across all variants. Figure 4 details the main 73-writer protocol and the supplementary 180-writer expanded check.

In addition to the original 73-writer (51/10/12) protocol, we rebuild a writer-disjoint split over full offline HWDB1.1 (180 writers; 125/27/28) and re-run k=5 fusion evaluation on 28 test writers for seeds 0 and 1 (directional consistency check; not a full five-seed claim). Absolute MAE is not interchangeable with the 12-writer table. As a modularity check, we also integrate FontRNN [29] as a second online trajectory generator (n=50 exported trajectories; same width+fusion stack).

### 3.2. Implementation Details

The width-predictor uses a BiLSTM backbone over 80-point trajectories with width-specific trajectory features. Generic models are trained on 51 training writers using Adam ($\mathrm{lr}=10^{-3}$, batch size 64) until validation MAE plateaus. Few-shot adaptation runs for 50 epochs on *k* support images per-writer with the same optimizer and anchor strength $\lambda =10^{-4}$. Fusion hyperparameters $\left(\beta_{\min},\beta_{\max},\mathrm{smooth}\_\mathrm{kernel}\right)$ are selected on 10 validation writers only. All experiments use a single NVIDIA GPU workstation; inference latency is measured on the same hardware for fair comparison.

### 3.3. Baselines and Evaluation Metrics

Baselines are grouped by experimental purpose rather than treated as a flat method list:

- **Baseline 1 (Generic prediction):** No writer adaptation; evaluates whether writer-specific adaptation is necessary.
- **Baseline 2 (Fixed-width support mean):** Writer-level thickness constant; evaluates strong population/writer priors.
- **Baseline 3 (Full fine-tuning, Conf FT):** Updates all parameters; evaluates overfitting risk under limited samples.
- **Baseline 4 (Parameter-efficient adaptation, PEFT):** Efficient writer conditioning used as the adaptation backend.
- **Baseline 5 (Fixed fusion):** Constant β blending after post-processing; evaluates whether adaptive weighting outperforms manually selected fixed-ratios.
- **Proposed (Dynamic fusion):** Confidence-guided per-point $\beta_{t}$ blending in the deployed rendering stack via a deterministic confidence-to-β map.

External diffusion baselines (One-DM, DiffBrush) are evaluated under the same writer-disjoint splits for reference [14,17]. One-DM and DiffBrush are image-generation baselines rather than drop-in trajectory width estimators; comparisons are complementary where input and adaptation conditions differ (Section 4.8).

**Primary endpoints** are Stage-B stability metrics on the deployed rendering stack: writer-level standard deviation, worst-writer error, cross-seed coefficient of variation, run-level win rate, and fused-stack point-wise MAE. We evaluate **two stages separately**: Stage-A reports model-only MAE (generic width/fixed support statistics without deployed blending); Stage-B reports deployed fusion-stack MAE after rule and model blending and post-processing (writer-adapted PEFT + fusion). The Stage-B output mixes learned widths with geometric rule priors for robustness under noisy pseudo-labels. Point-wise MAE is a geometric fidelity measure, not a complete personalization score; complementary style-preservation metrics are reported later.

Auxiliary metrics include Traj-TV, high-frequency width energy, PSNR, and writer cosine. Imaging perturbations (blur, brightness, JPEG) test confidence degradation under input corruption. **Security-oriented analysis** reports attack success rate (ASR) at 0.1/1/5% false-accept rate (FAR) using Shallow CNN, ResNet-18, and HOG+SVM as a diagnostic of detector sensitivity, not an end-to-end spoofing claim [22,23,24,30]. Run-level 2.5/97.5% percentiles of fused MAE quantify writer-seed heterogeneity (reported with the later run-percentile table).

## 4. Experiments

### 4.1. Fusion-Stack Evaluation (Primary)

Our primary comparison is restricted to the *deployed fusion-stack*: rule-only, model-only (adapted), fixed fusion, and dynamic fusion outputs used for rendering. Stage-A predictor baselines (generic model, fixed-width support-mean) are deferred to Appendix A because they measure pre-fusion width estimates rather than the stack actually deployed at render time.

**Figure 5:**
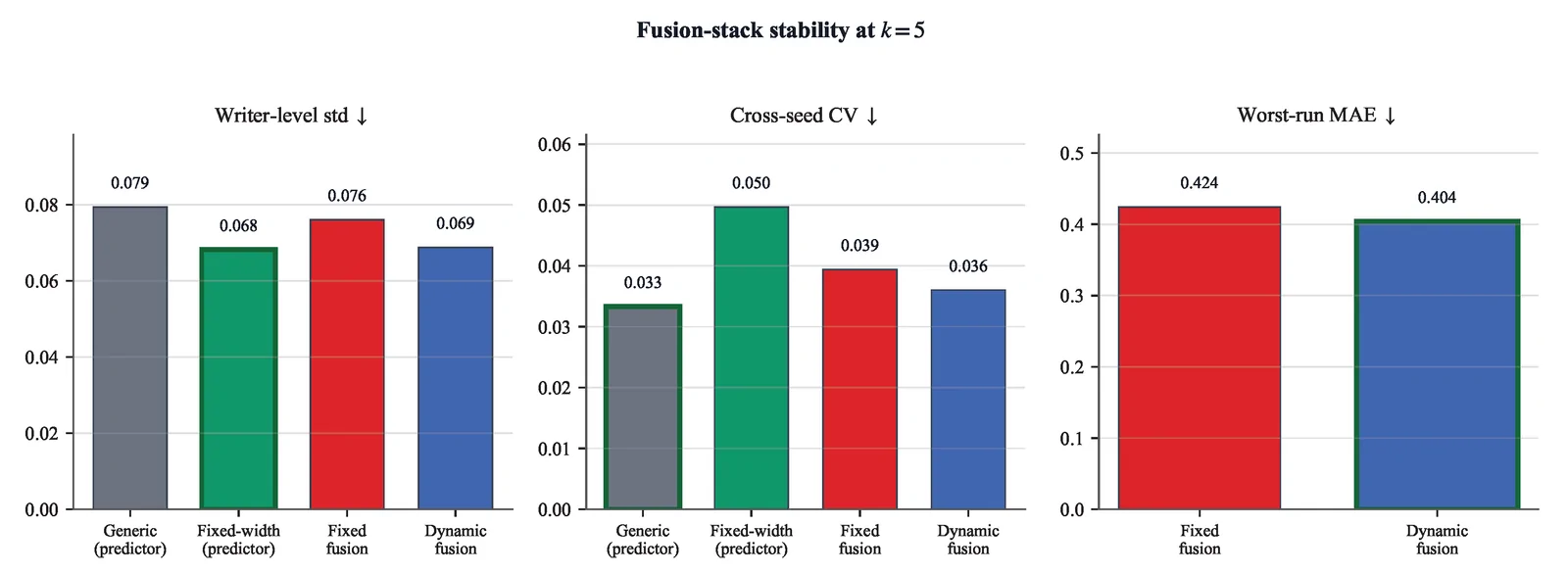
Primary stability metrics at k=5: dynamic fusion yields the lowest writer-level std and cross-seed CV; The right subgraph compares worst-run fused-stack error (green border = best within panel). The 12 held-out test writers are under the 73-writer writer-disjoint split.

**Figure 6:**
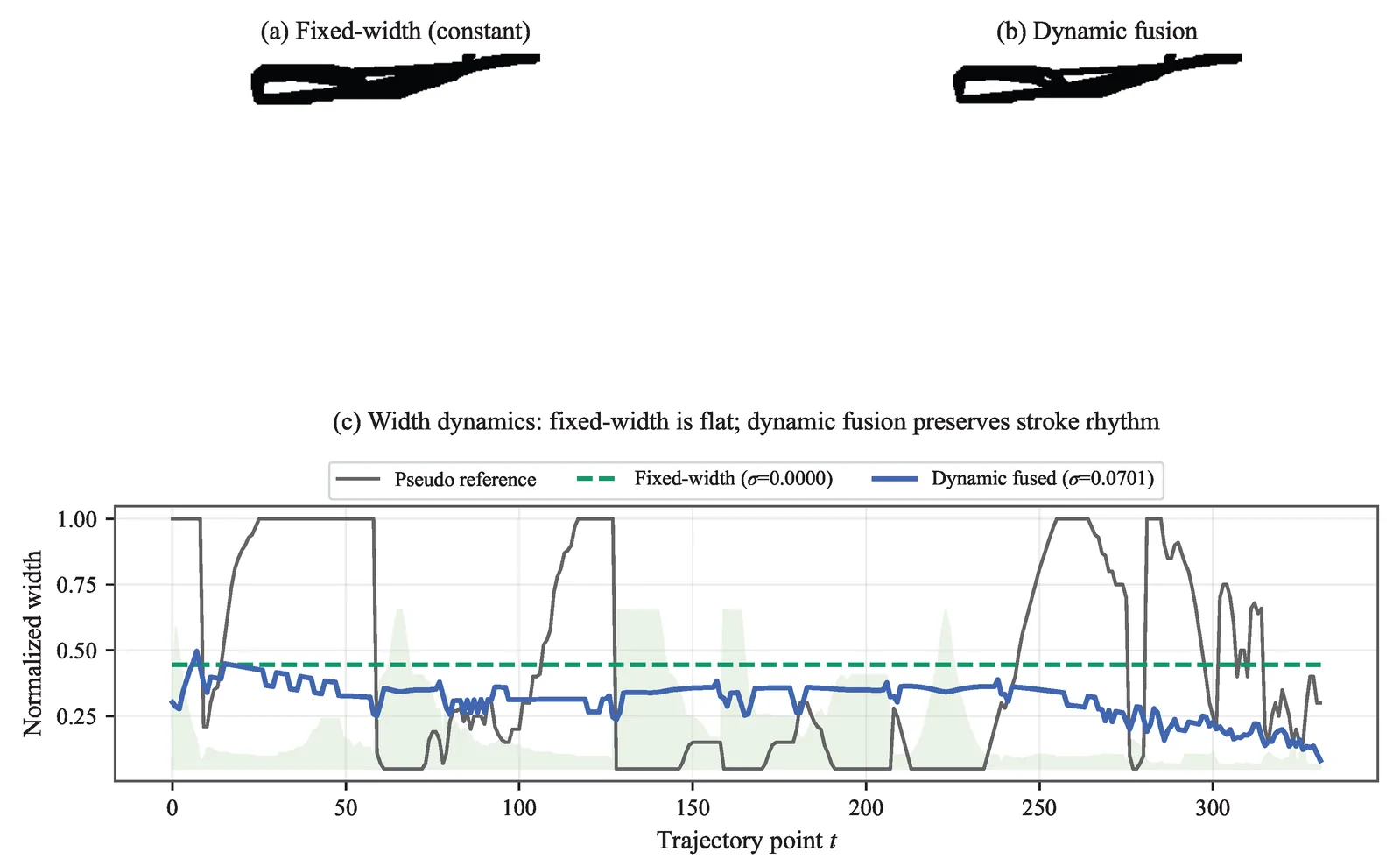
Fixed-width *predictor* (Appendix A) vs. dynamic *fusion* on the same character. (**a**) Fixed-width render: constant thickness (σ≈0). (**b**) Dynamic fusion render: writer-specific rhythm preserved. (**c**) Width profiles: green dashed = constant predictor; blue = dynamic fused output; black = pseudo reference.

**Figure 7:**
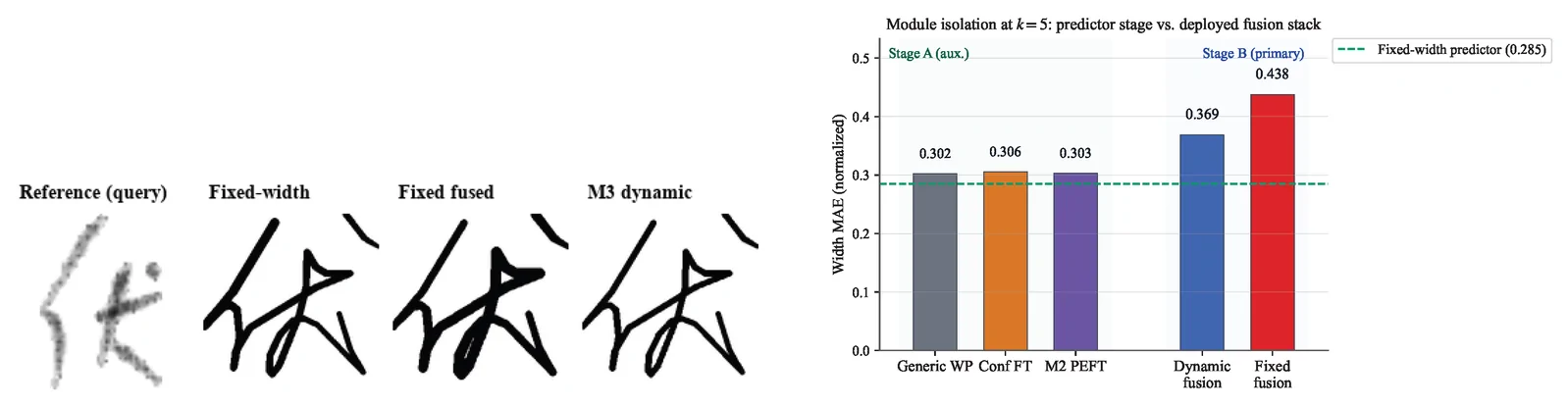
(**a**) Rendered width variants on a test character (Chinese HWDB samples as study content). (**b**) Stage-wise diagnostics at k=5: Stage B bars are primary run-level fused-stack MAE (dynamic 0.292/fixed 0.303; see the primary fusion-stack tables); Stage A predictor MAE is auxiliary (Appendix A). The 12 held-out test writers are under the 73-writer writer-disjoint split.

Table 1 summarizes stability on the fusion-stack at k=5 over 60 writer-disjoint runs (12 test writers × 5 seeds). Dynamic fusion wins 54 of 60 run-level comparisons, improving both stability (writer std, worst-writer error, cross-seed CV) and mean MAE relative to fixed fusion (Table 1). It also outperforms the generic Stage-A baseline on the deployed-stack (0.292 vs. 0.302; Appendix A). Figure 5 visualizes the stability advantage; Figure 6 contrasts fixed-width prediction with dynamic fusion on the same character; Figure 7 isolates stage-wise behavior.

### 4.2. Stratified Reliability Analysis

Because reliability imbalance varies across writers and stroke regions, we stratify the primary fusion comparison along two axes without changing the writer-disjoint protocol.

**Figure 8:**
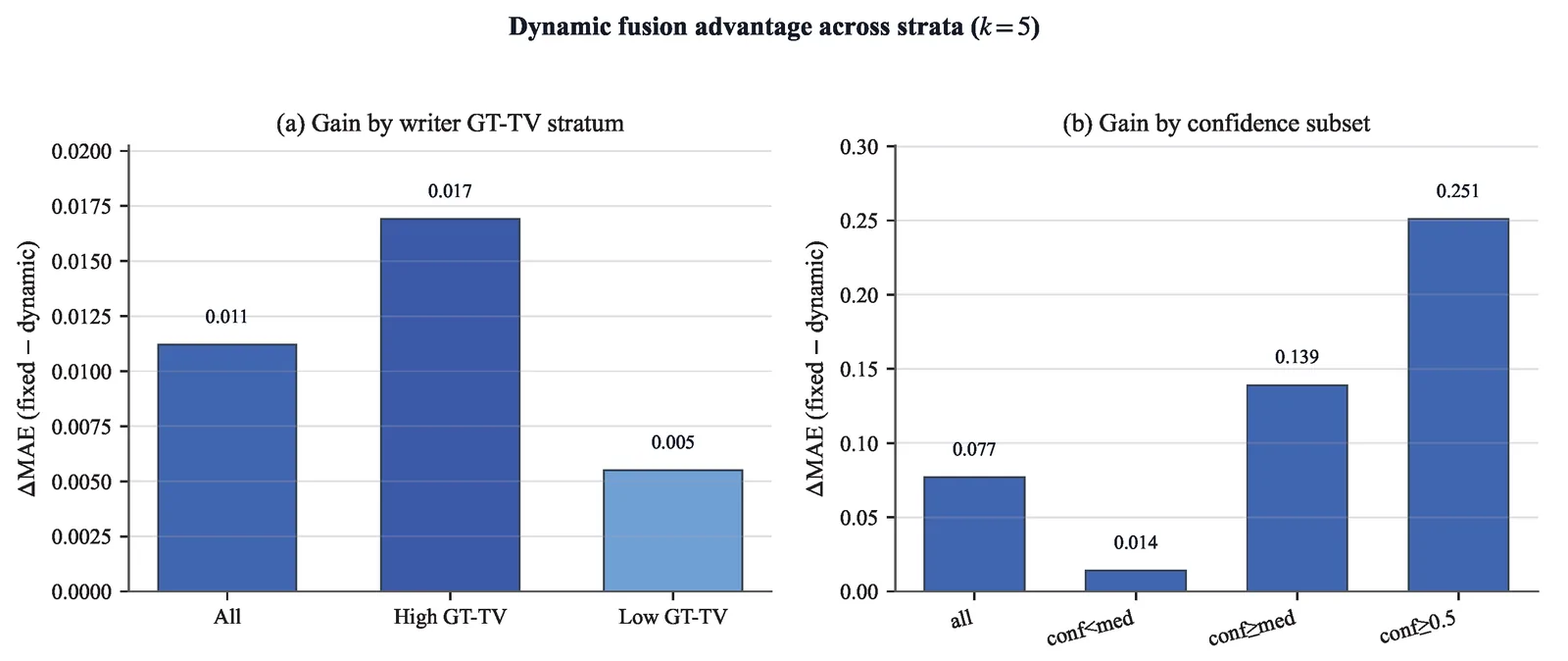
Stratified dynamic versus fixed fusion gain at k=5: by writer GT-TV stratum (**a**) and by confidence subset (**b**). The 12 held-out test writers are under the 73-writer writer-disjoint split.

**Writer thickness variability.** Test writers are split by median ground-truth width temporal variation (TV) computed on held-out queries. Table 2 shows that dynamic fusion outperforms fixed fusion in **30/30 high-GT-TV runs** and **24/30 low-GT-TV runs**, with larger absolute MAE gain in the high-variability group (0.017 vs. 0.006). Figure 8 summarizes the stratum-wise gains.

**Confidence-subsets.** We additionally report pooled fused-stack MAE on trajectory points split by pseudo-label confidence (Table 3). Dynamic fusion maintains a positive margin on every subset; the gain increases from 0.014 on low-confidence points (conf < median) to 0.139 on high-confidence points (conf ≥ median) and 0.251 on the top-confidence band (conf ≥ 0.5), consistent with reliability-guided weighting being most effective where pseudo-labels are trustworthy.

### 4.3. Analysis of Reliability Adaptation

**Figure 9:**
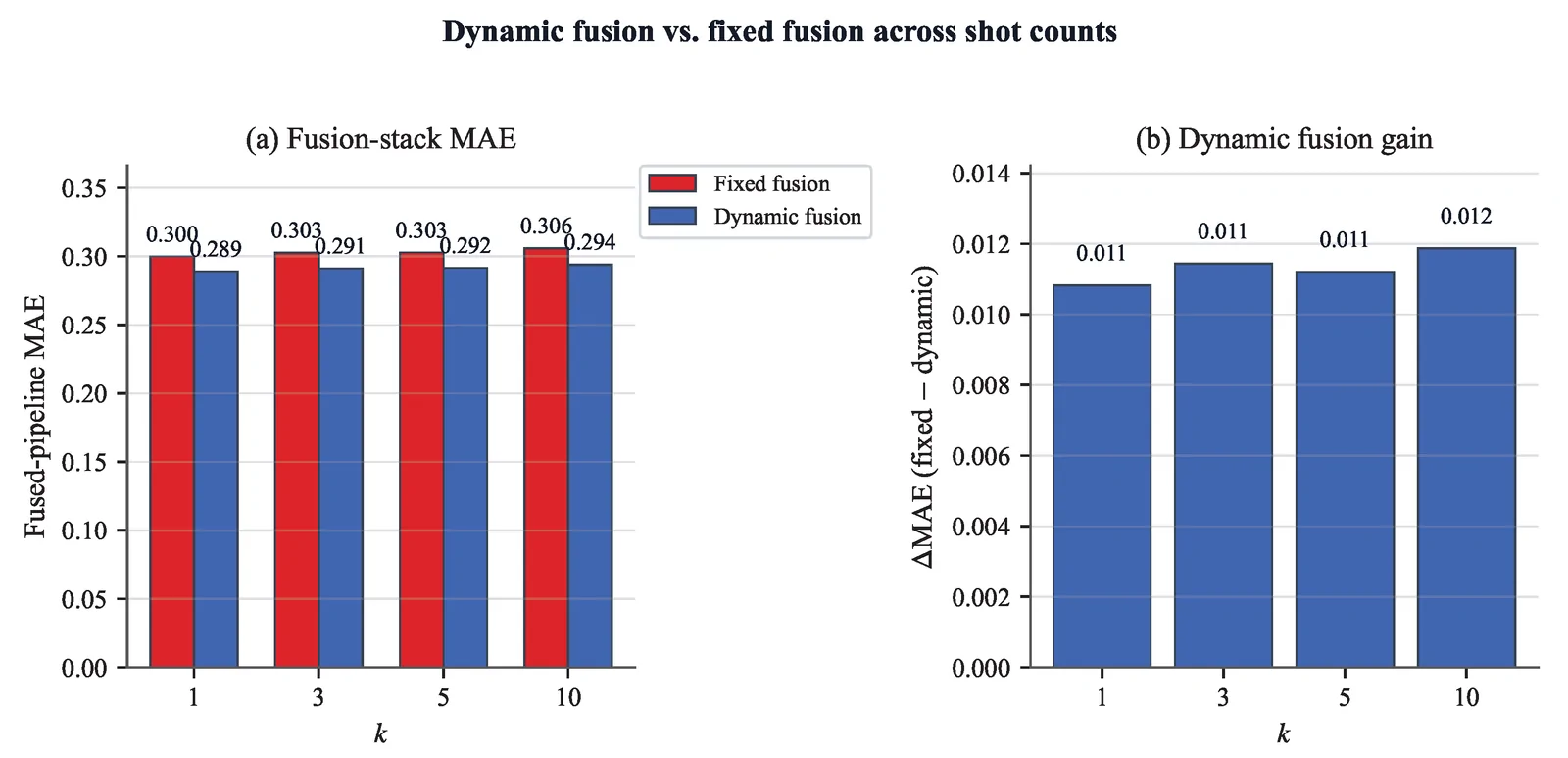
Dynamic fusion gain over fixed blending across shot counts (Δ = fixed − dynamic MAE). The 12 held-out test writers are under the 73-writer writer-disjoint split.

**Figure 10:**
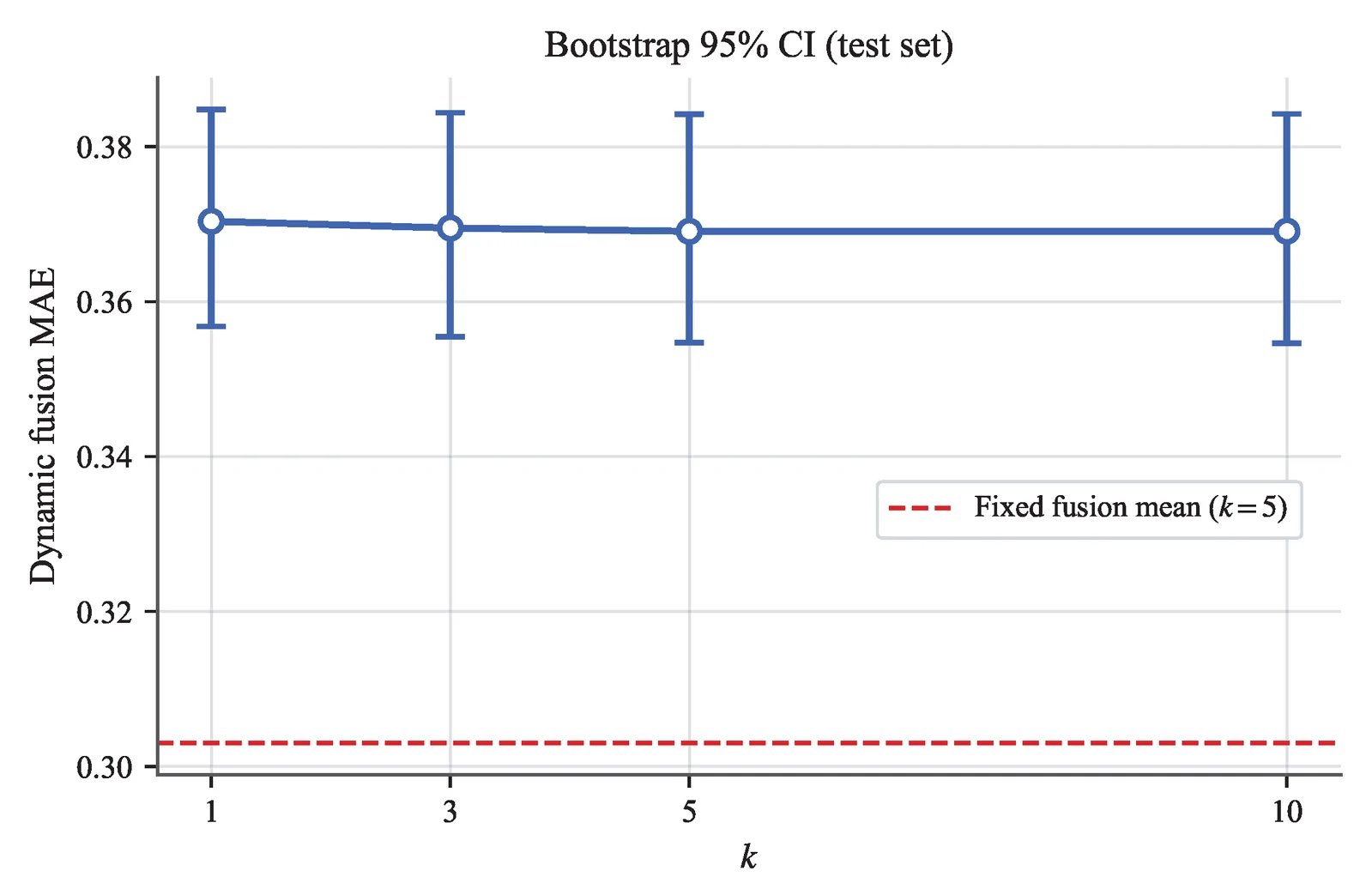
Run-level dynamic fusion MAE (markers) with 2.5/97.5% run percentiles (error bars); dashed line: fixed-fused mean at k=5 (0.303). Primary means match Table 4 and Table 5. The 12 held-out test writers are under the 73-writer writer-disjoint split.

To verify whether adaptive reliability estimation is beneficial, we compare the proposed dynamic fusion strategy with fixed-ratio fusion under identical experimental settings. Fixed fusion assumes constant source reliability; the observed improvement of dynamic fusion (0.303 → 0.292 MAE at k=5; Table 4, Figure 9) indicates that reliability varies across writers and requires adaptive weighting. Absolute gains are modest; paired run- and writer-level tests after Table 4 quantify significance against model-only and validation-selected fixed β=0.9. The gain is stable for k∈{1,3,5,10} (Δ≈0.011–0.012; Table 4), and the run-level 95% interval for dynamic MAE at k=5 is [0.172, 0.399] (2.5/97.5% percentiles over the 60 writer-seed runs; Table 5, Figure 10); the interval is wide because it reflects writer-level heterogeneity rather than a tight CI of the mean alone. Figure 3 shows that $\beta_{t}$ co-varies with confidence along the stroke.

**Figure 11:**
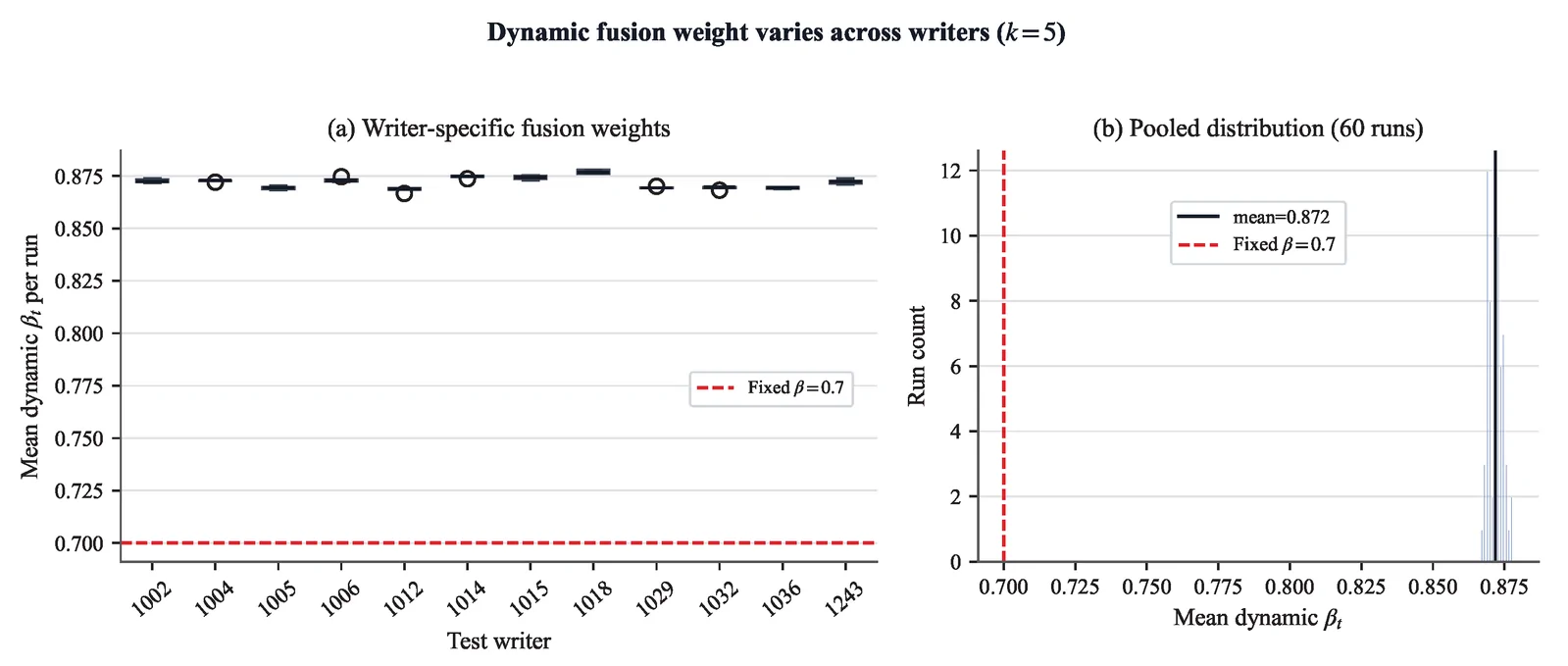
Cross-writer dynamic-β distribution at k=5 (illustrative panels; reported MAE uses $\beta_{t}\in \left[0.85,0.99\right]$). (**a**) Per-writer box plots over five seeds (dashed: illustrative fixed reference β=0.7). (**b**) Pooled run-level histogram (60 runs). The 12 held-out test writers are under the 73-writer writer- disjoint split.

**Figure 12:**
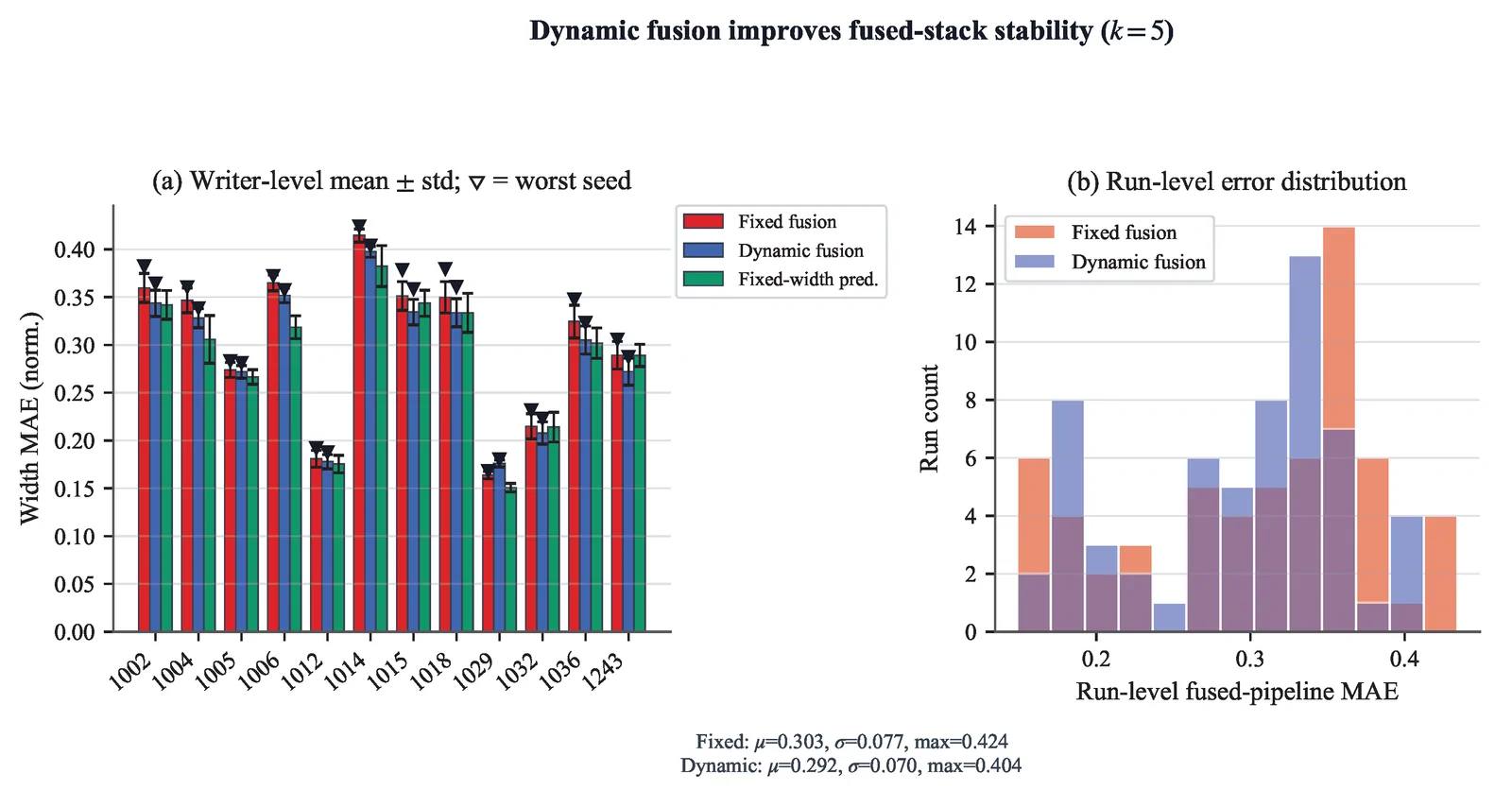
Error stability analysis at k=5. (**a**) Writer-level mean±std over seeds; downward markers indicate worst seed. (**b**) Run-level MAE histogram for fixed vs. dynamic fusion (60 runs). The 12 held-out test writers are under the 73-writer writer-disjoint split.

**Figure 13:**
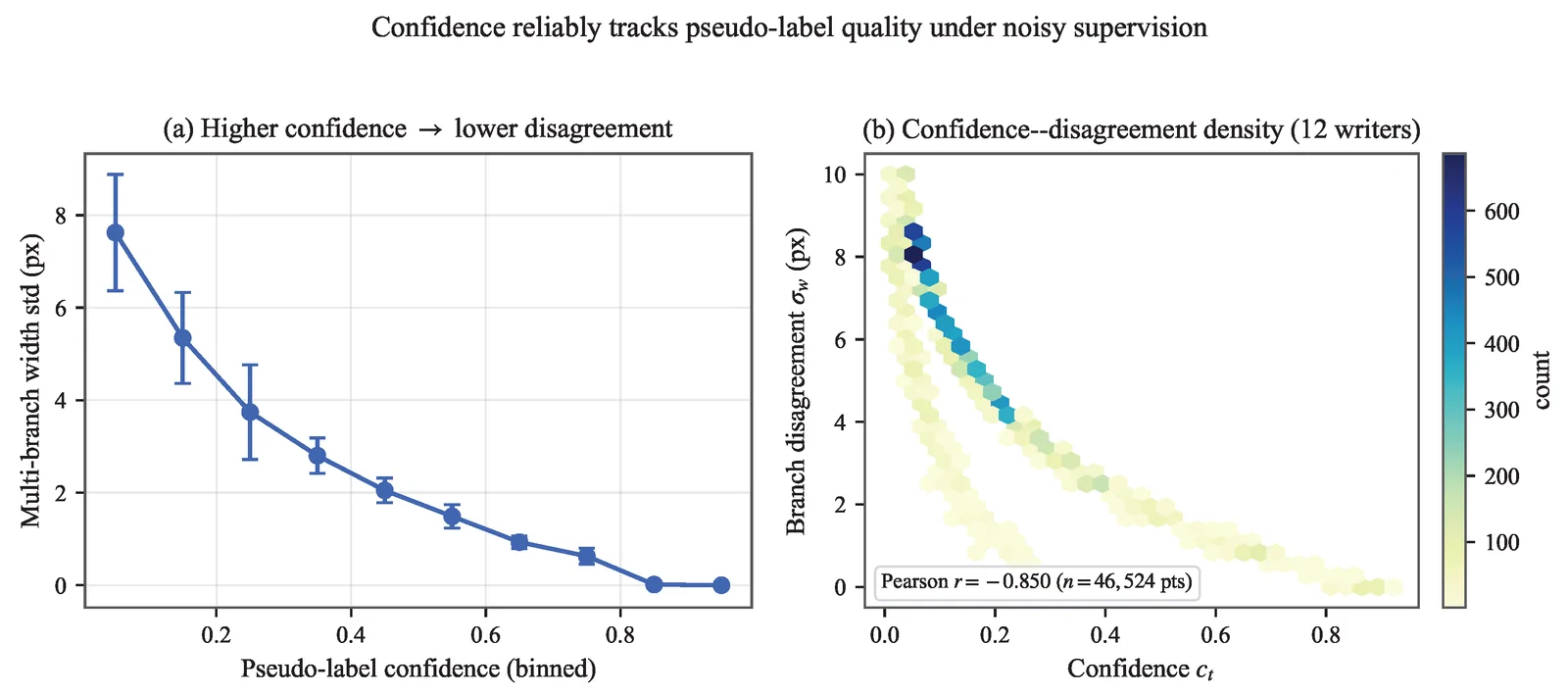
Confidence and disagreement validation on 12 held-out writers (k=5, seed 0): binned multi-branch width std decreases with confidence (**a**); point-wise density and Pearson correlation (**b**). The 12 held-out test writers are under the 73-writer writer-disjoint split.

**Figure 14:**
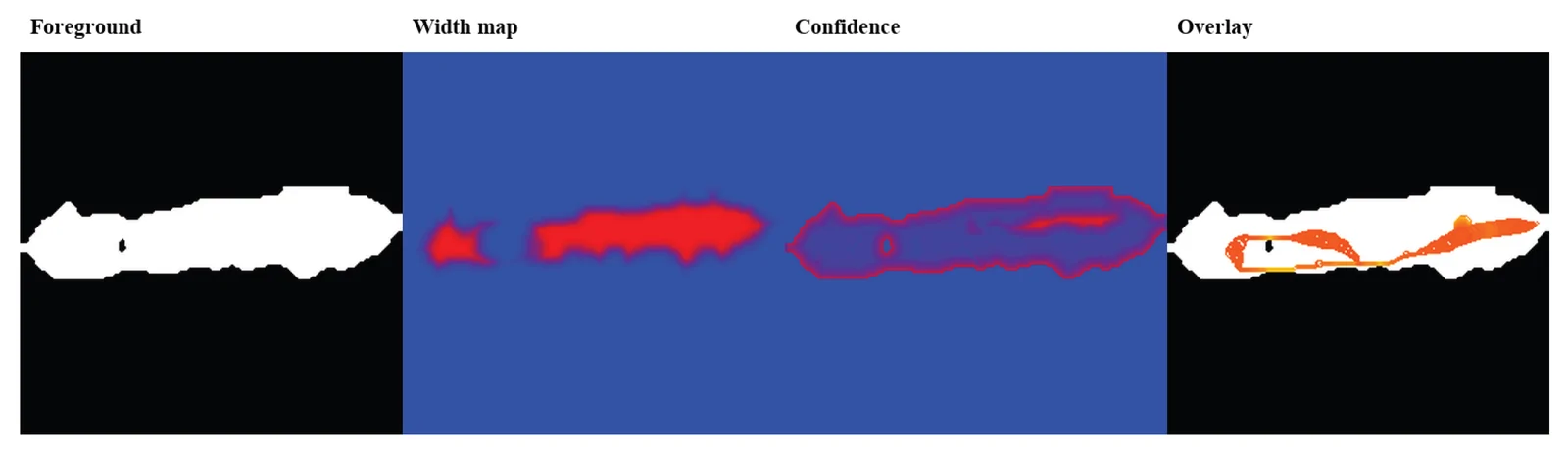
Spatial reliability of pseudo-labels: foreground, width map, confidence map, and skeleton overlay.

**Figure 15:**
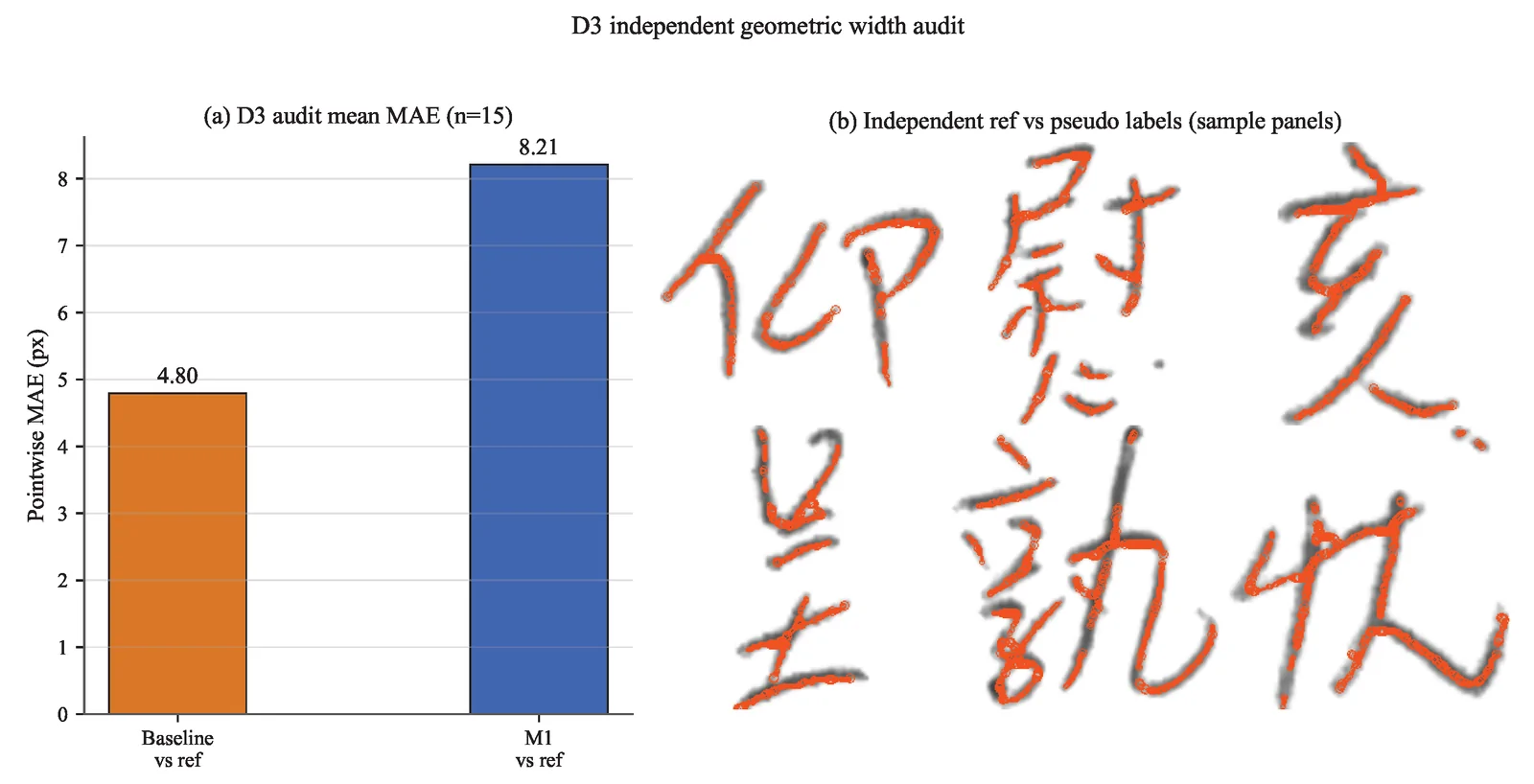
Independent width audit (15 images): pseudo-labels deviate from a separate geometric reference; confidence weighting avoids treating all points equally. Chinese HWDB character images are the study content (not UI text).

**Figure 16:**
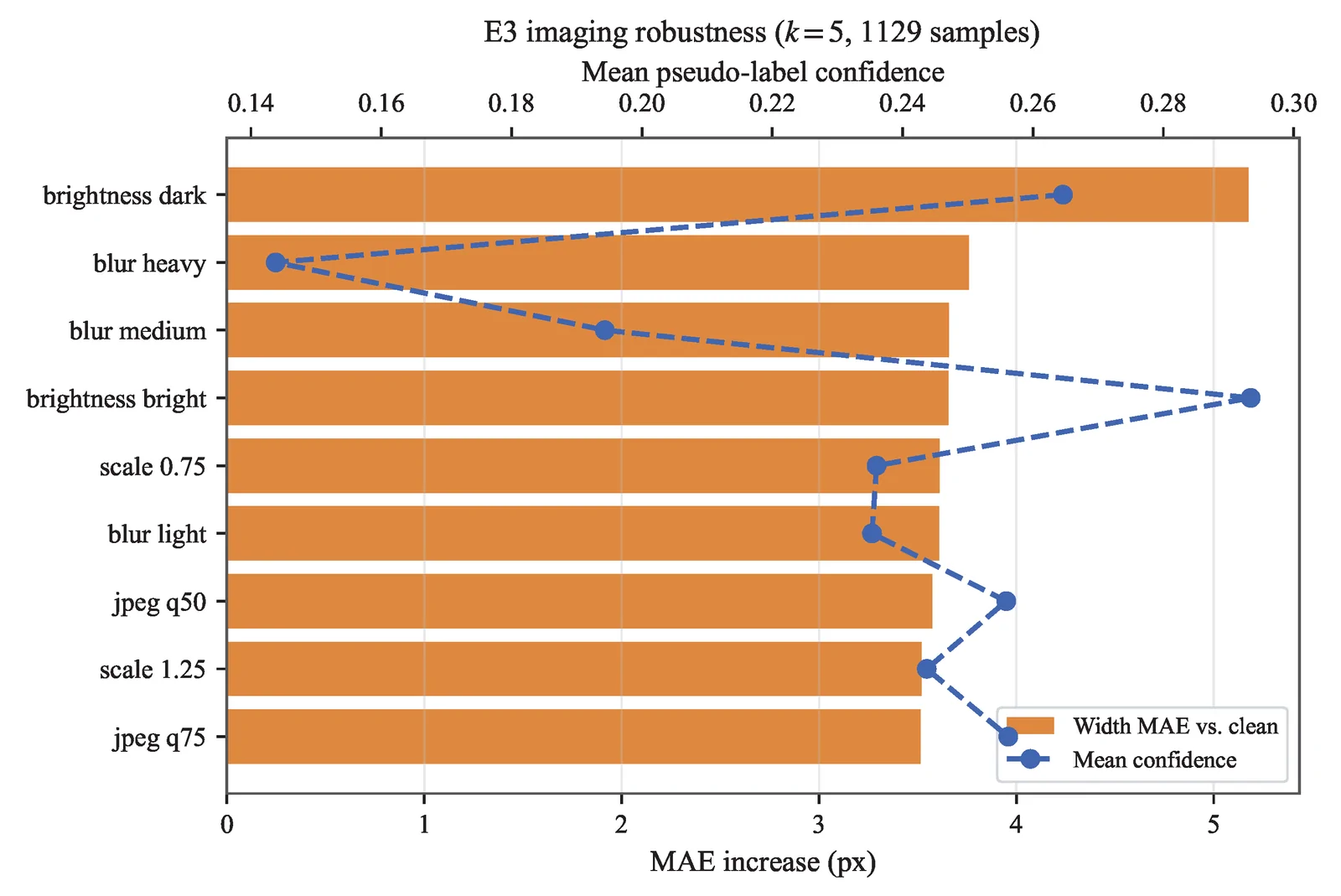
Imaging robustness: width error and mean confidence degrade together under blur and brightness perturbations.

**Figure 17:**
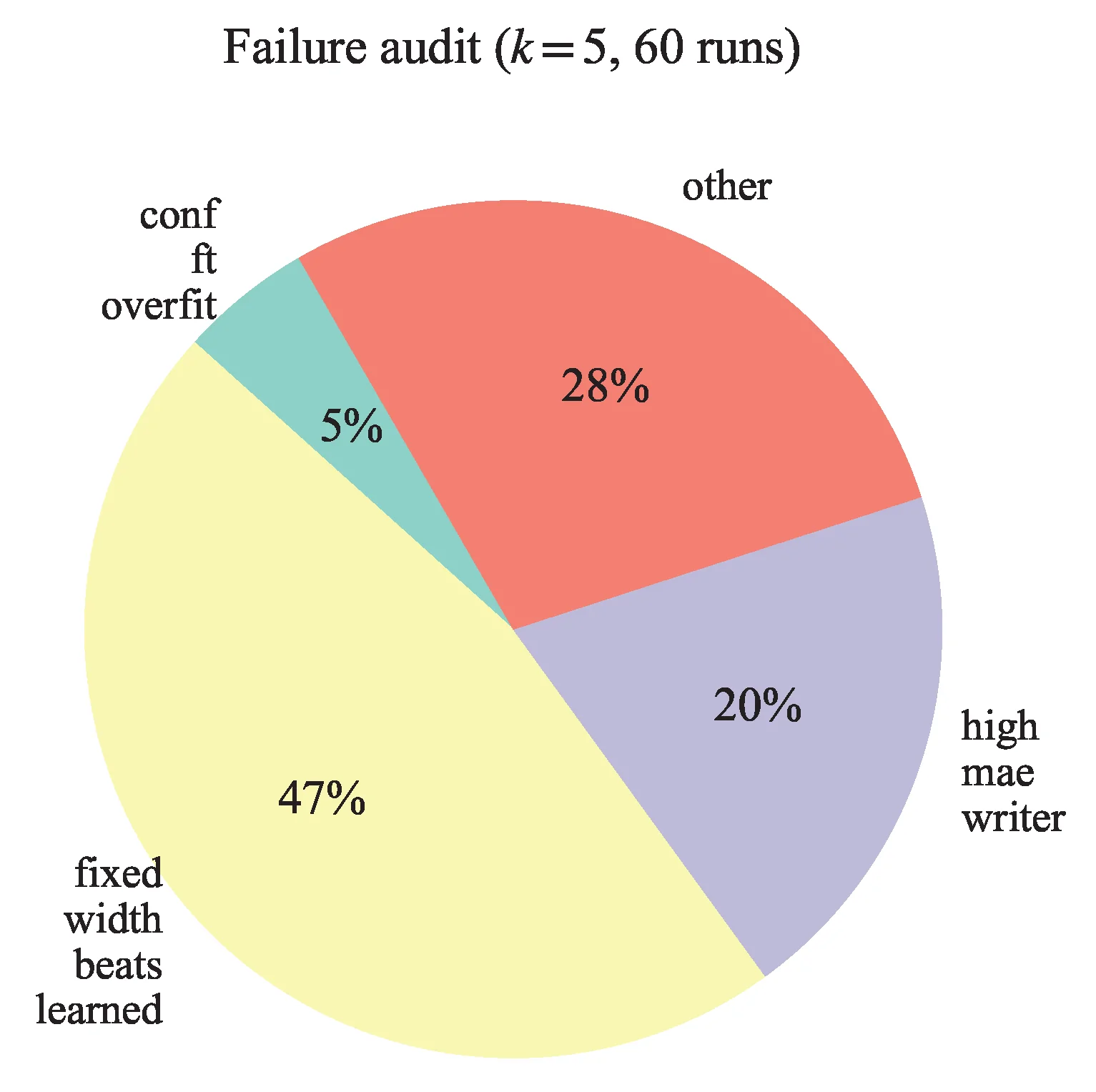
Writer-level error audit (k=5, 60 runs): per-run categories when fixed-width support-mean beats generic by >0.01 MAE or adaptation overfits. The 12 held-out test writers are under the 73-writer writer-disjoint split.

To understand why dynamic fusion outperforms fixed fusion, we examine the learned fusion weights $\beta_{t}$ across different writers. Reported fused MAE uses the validation-selected clip $\left(\beta_{\min},\beta_{\max}\right)=\left(0.85,0.99\right)$ (Table 4). Figure 11a shows per-writer box plots of $\beta_{t}$ at k=5; writer-level medians still differ within the clipped operating range, so a single manually chosen fixed coefficient cannot match all writers equally well (dashed line: illustrative fixed reference β=0.7). Figure 11b presents the pooled histogram over 60 runs, confirming that no single β value is optimal for all writers. This writer-dependent variation in fusion weights directly supports our central premise: reliability of different width sources is not uniform, and adaptive combination is necessary for robust personalization. Figure 12 and Table 1 further show reduced run-level variance and worst-case error under dynamic fusion. Figure 13 validates that higher pseudo-label confidence correlates with lower multi-branch width disagreement (strong negative Pearson correlation over $∼4\times 10^{4}$ trajectory points from 12 writers), supporting the reliability signal used for dynamic $\beta_{t}$. Reliability imbalance manifests spatially in confidence maps (Figure 14). An independent geometric width audit on 15 held-out images confirms noisy pseudo-labels (∼5 px mean deviation; Figure 15), and imaging perturbations co-degrade confidence and width error (Figure 16). Across-writer failure modes are summarized in Figure 17.

Paired significance at k=5 supports the modest absolute gain. Dynamic fusion (≈0.292) beats model-only (β=1, ≈0.303) on 54/60 runs ($p\;\approx \;2.7\times 10^{-9}$, $d_{z}\;\approx \;1.19$) and beats validation-selected fixed β=0.9 (≈0.295) on 55/60 runs ($p\;\approx \;1.6\times 10^{-10}$, $d_{z}\;\approx \;1.58$). At the writer level, dynamic fusion is better than validation-selected fixed β=0.9 on 11/12 writers (Wilcoxon p≈0.0015) and better than model-only on 11/12 (p≈0.0034). A writer-bootstrap of the dynamic mean MAE yields 0.292 with 95% CI ≈[0.253,0.329]. Absolute gains remain modest; we therefore moderate claims accordingly and report both run-level and writer-level aggregation.

**Fixed-β grid with validation selection.** We report an explicit fixed-β grid with the same post-processing stack as dynamic fusion (stroke split, optional smoothing kernel 3, identical rendering): β∈{0.50,0.55,…,1.00} (step 0.05). Validation selects the minimum run-mean MAE at β=0.90 (val MAE 0.2511), coinciding with the paper’s operating point; the validation curve is flat near the optimum (β=0.85/0.90/0.95 give 0.2514/0.2511/0.2513), and dynamic fusion on validation reaches 0.2501. On the held-out test set with β frozen from validation, dynamic fusion attains 0.2916 vs. fixed β=0.90 at 0.2948 (55/60 runs; $p\;\approx \;1.6\times 10^{-10}$, $d_{z}\;\approx \;1.58$; 11/12 writers, p≈0.0015). Lower β can yield lower test MAE if selected post hoc (e.g., β=0.50→0.280), but that would be test-set tuning; we do *not* promote post-hoc β=0.50 as the official baseline. The fair comparison is against the validation-selected β=0.90, which dynamic fusion still beats.

### 4.4. Few-Shot Adaptation Analysis

**Figure 18:**
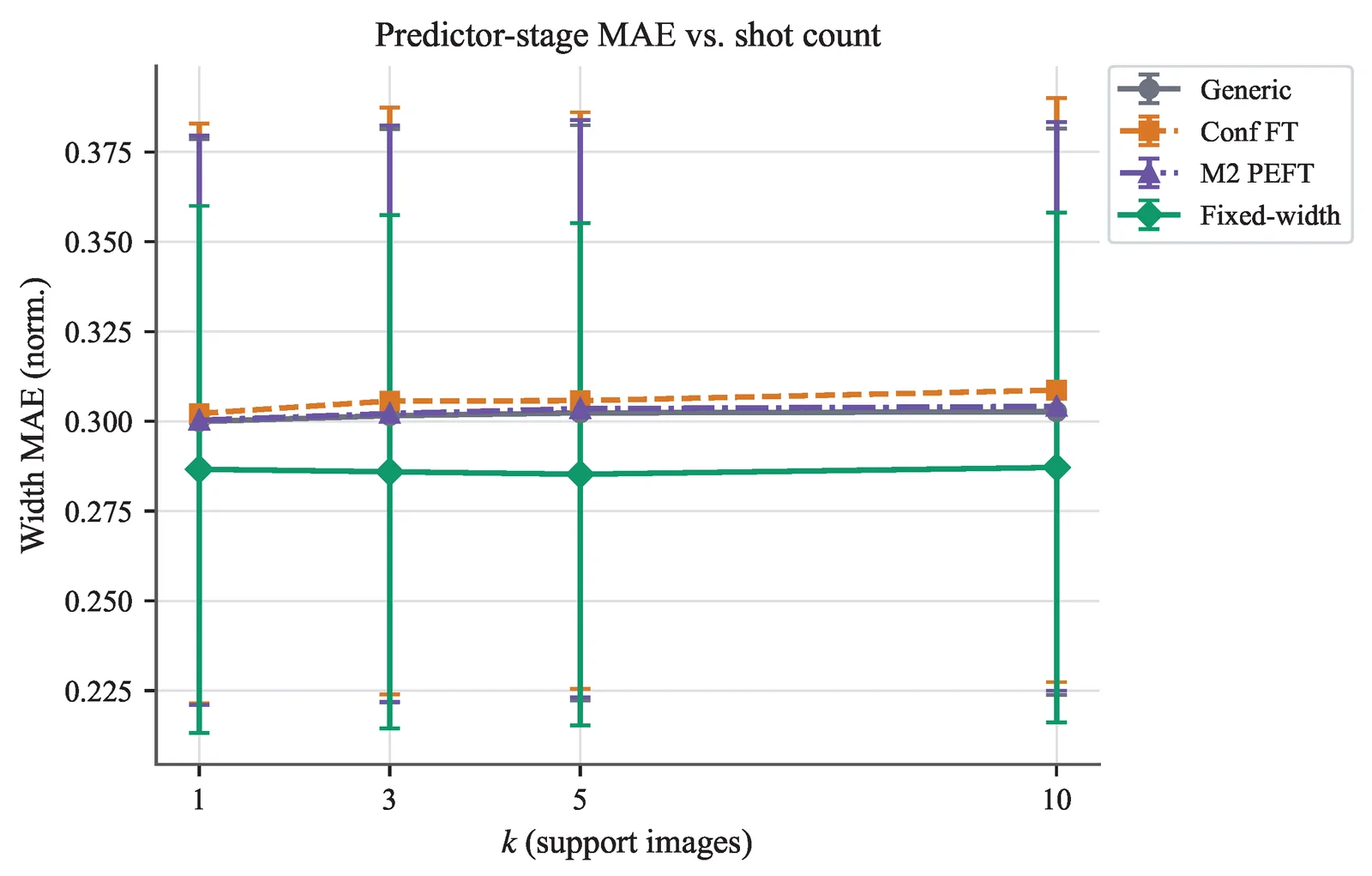
Per-point width MAE vs. shot count *k*. Predictor variants remain near 0.30; dynamic fusion gain over fixed blending is stable across *k*. The 12 held-out test writers are under the 73-writer writer-disjoint split.

**Figure 19:**
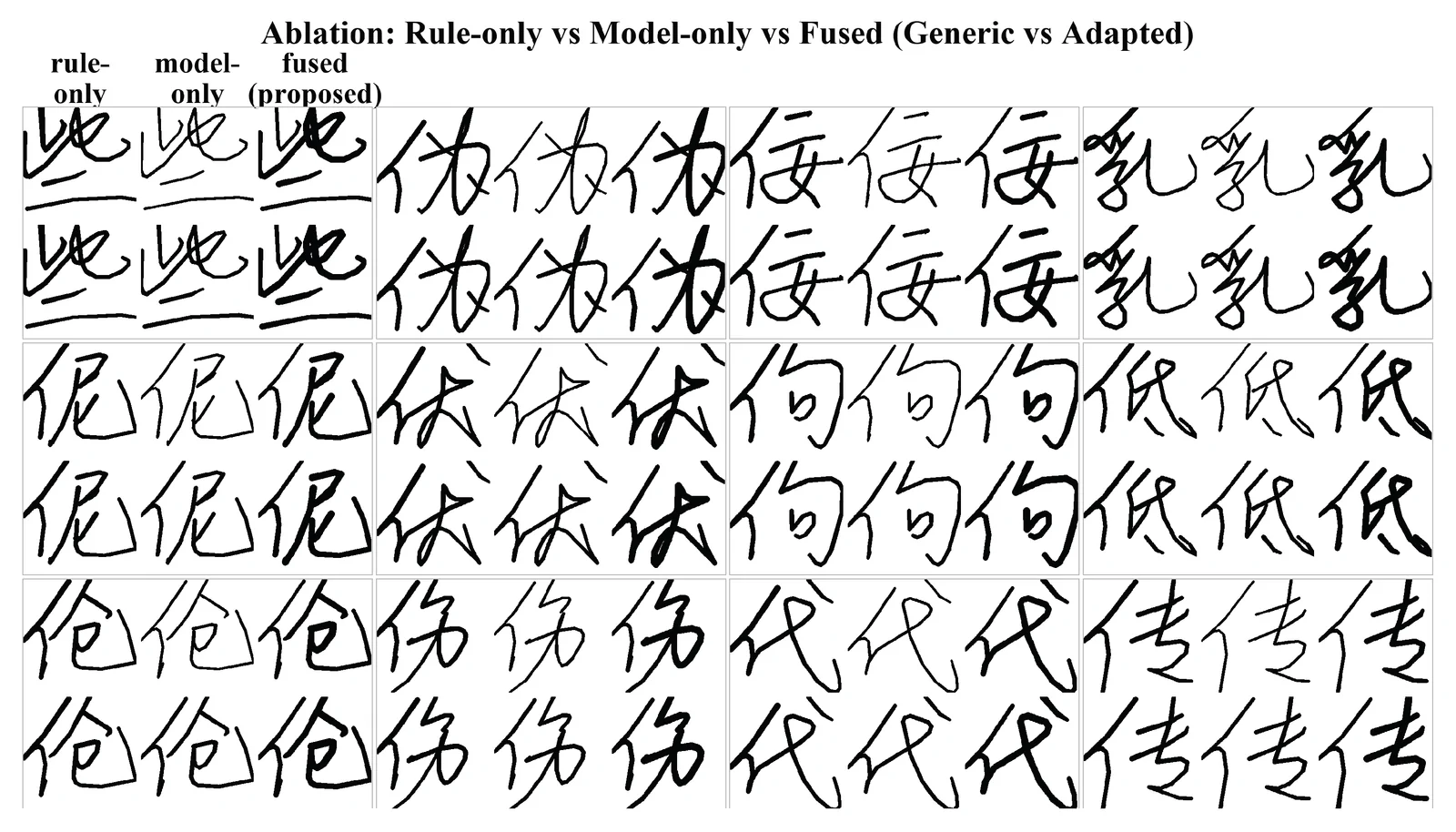
Qualitative rendering ablation on SDT-generated characters (my_style; Chinese HWDB-style samples): generic vs. adapted rows; rule-only/model-only/fused columns.

Figure 18 plots per-point MAE vs. shot count *k*. Generic, Conf FT, and PEFT cluster near MAE 0.30 across *k*, suggesting that additional support primarily affects adaptation stability rather than dramatically shifting average predictor error. By contrast, dynamic fusion maintains stable fused-pipeline performance as *k* decreases (Table 4), demonstrating suitability for few-shot scenarios where support is extremely limited. In 28 of 60 runs at k=5, the fixed-width support-mean predictor beats the generic predictor by more than 0.01 MAE (Figure 17), illustrating that lower Stage-A point error does not imply better personalization; qualitative rendering (Figure 6 and Figure 19) shows that fused outputs preserve writer-specific thickness variation whereas fixed-width remains visually flat. A complementary style-reference benchmark on single-writer photos (my_style; Appendix C) yields dynamic-over-fixed fusion gains of +0.28 to 0.34 MAE under both direct-photo and SDT-pipeline protocols, reinforcing the fusion contribution on homogeneous thickness references.

### 4.5. Manual Stroke-Width Audit

To address circular evaluation concerns, we conducted a manual stroke-width annotation study on the 12 test writers: 871 matched click points (match distance ≤12 px on the 256×256 canvas). Manual widths are never used as PEFT training targets. **Scale caveat.** Click mean ≈15.1 px vs. model branch ≈2.2 px and rule branch ≈5.0 px; both branches are thinner than clicks, so any convex fusion remains between them and absolute-pixel MAE favors the thicker (rule) branch. We therefore treat manual labels strictly as an audit of circularity and scale—not as a fair primary ranking of fusion under the large-scale pseudo-label protocol—and also report leave-one-writer-out (LOWO) calibrated MAE (Table 6). Main conclusions do *not* depend on manual rankings.

Writer-level paired tests (12 writers): on raw MAE, dynamic vs. rule is better on 0/12 writers ($p\;\approx \;4.9\times 10^{-4}$), as expected under absolute-scale mismatch; on calibrated MAE, dynamic vs. rule is better on 3/12 (p≈0.30, not significant). On the same audit, dynamic still beats fixed (12/12 raw and calibrated, $p\;\approx \;4.9\times 10^{-4}$) and model-only (12/12 raw; 11/12 calibrated). The audit’s scientific value is to expose that (i) pseudo-labels are not independent ground truth and (ii) learned gates collapse toward rule-like predictions under clicks; pseudo-label MAE remains the primary comparable protocol.

### 4.6. Learned Gating Networks (Optional Enhancement)

We additionally train lightweight learned gates on the 10 validation writers (pseudo-label supervision) and evaluate on held-out test writers (no test labels used for training). Variants include constant, linear-on-confidence, MLP-on-confidence, MLP-on-all-cues, and a wider MLP; seeds {0,1,2}. **Positioning.** Deterministic confidence-guided fusion remains the default, training-free operating point (inspectable and aligned with confidence ablations). Learned gates are an *optional data-driven enhancement* when extra fusion training and validation selection are acceptable; we do *not* claim the deterministic map is superior on every metric. Table 7 reports multi-seed pseudo-label test MAE on the 12-writer protocol: all learned kinds beat the deterministic map here, and the gap is stable.

On the expanded 28-writer pool (zero-shot; gates not retrained), the same HWDB-val-trained gates still undercut dynamic on pseudo MAE (e.g., seed 0: mlp_all 0.324±0.003 vs. dynamic 0.476; seed 1: 0.342±0.003 vs. 0.483; all 3/3 seeds beat dynamic). Under a FontRNN generator swap, however, zero-shot transfer of the same HWDB-trained MLP gates saturates mean β→1.0 (model-only collapse), and relative-to-rule MAE worsens vs. dynamic (gate ≈0.292 vs. dyn 0.288). Thus the learned-gate advantage on the HWDB pseudo-label protocol does not automatically transfer under a generator change, whereas the deterministic map remains defined without re-training. We therefore keep deterministic fusion as the default and report learned gates fully—including where they win—as an optional enhancement.

### 4.7. Expanded Writer Pool and Generator Modularity

On the expanded HWDB1.1 split (180 writers; 125/27/28; k=5; seeds 0–1 only), results are directionally consistent with the 12-writer primary tables (Table 8). Absolute MAE is not interchangeable with the 73-writer/12-test-writer protocol. For FontRNN modularity (n=50), relative-to-rule MAE is FontRNN dyn 0.287/fixed 0.281 and SDT (first 50) dyn 0.306/fixed 0.304; this is a modularity check only (GT=rule biases toward the rule prior), so we do not claim dynamic beats fixed from these numbers.

### 4.8. Security-Oriented Analysis

**Figure 20:**
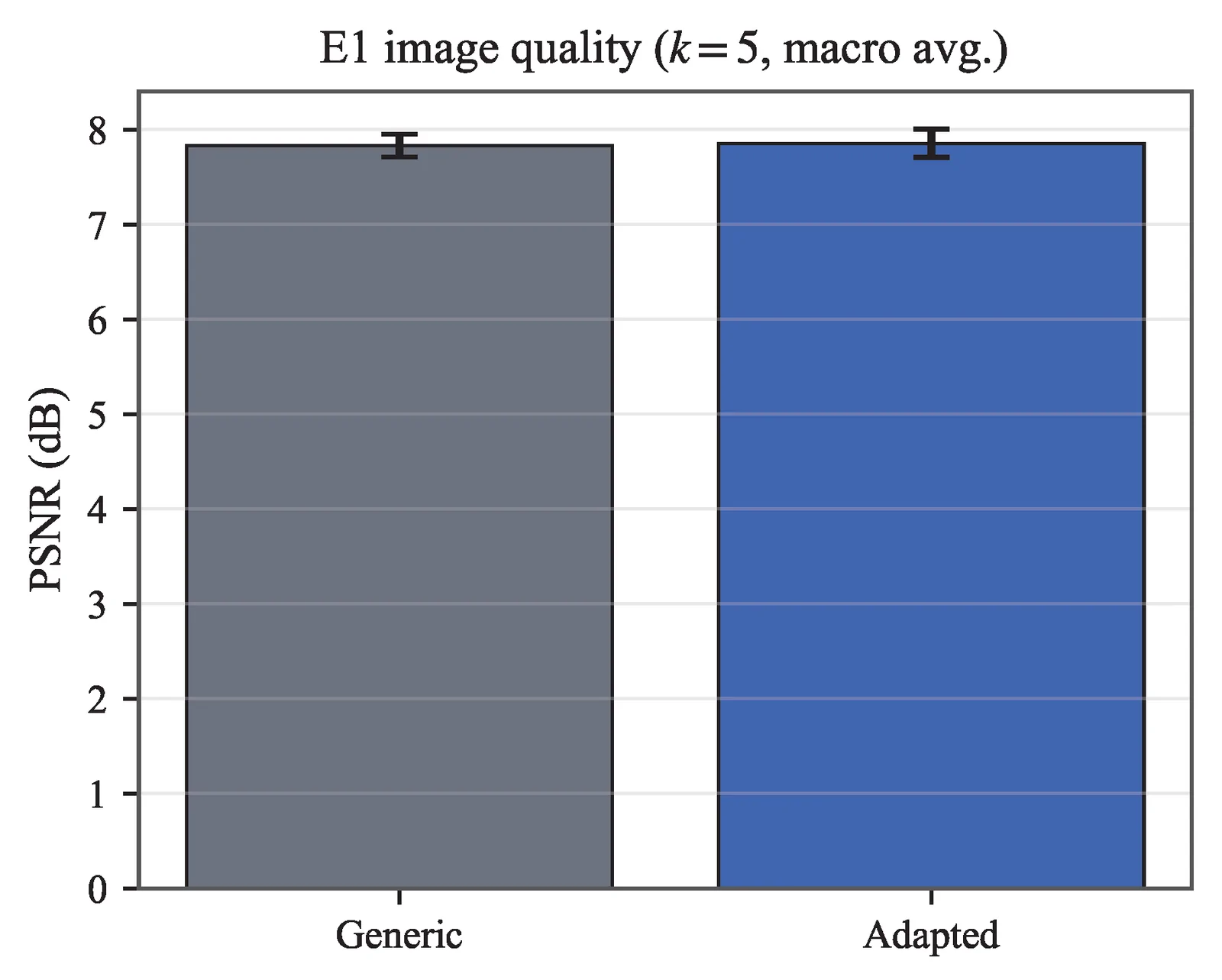
Rendered appearance (PSNR) remains comparable between generic and adapted-fused outputs. The 12 held-out test writers are under the 73-writer writer-disjoint split.

**Figure 21:**
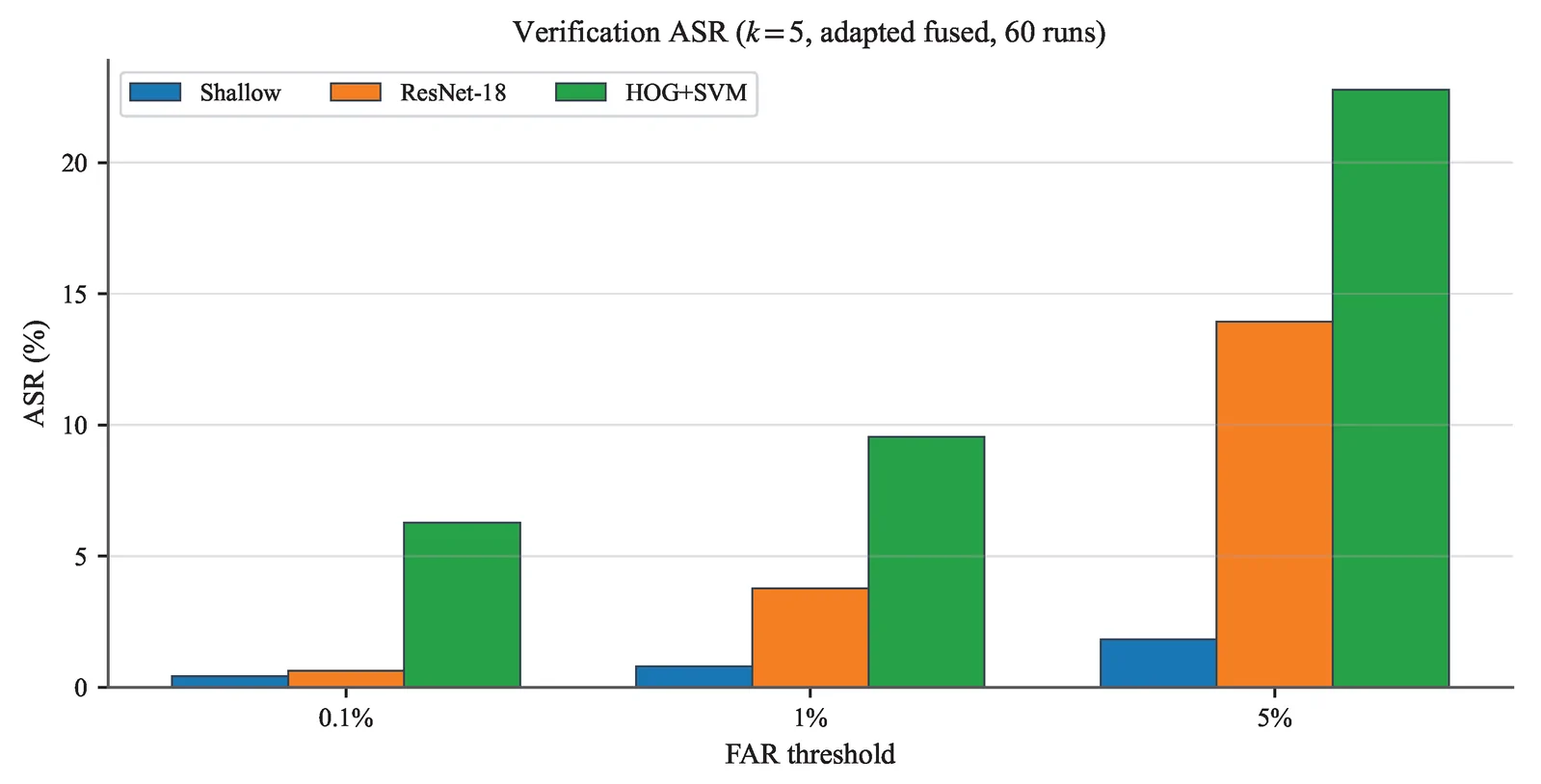
Security-oriented analysis: verification response at three FAR levels (adapted-fused, k=5). The 12 held-out test writers are under the 73-writer writer-disjoint split.

**Figure 22:**
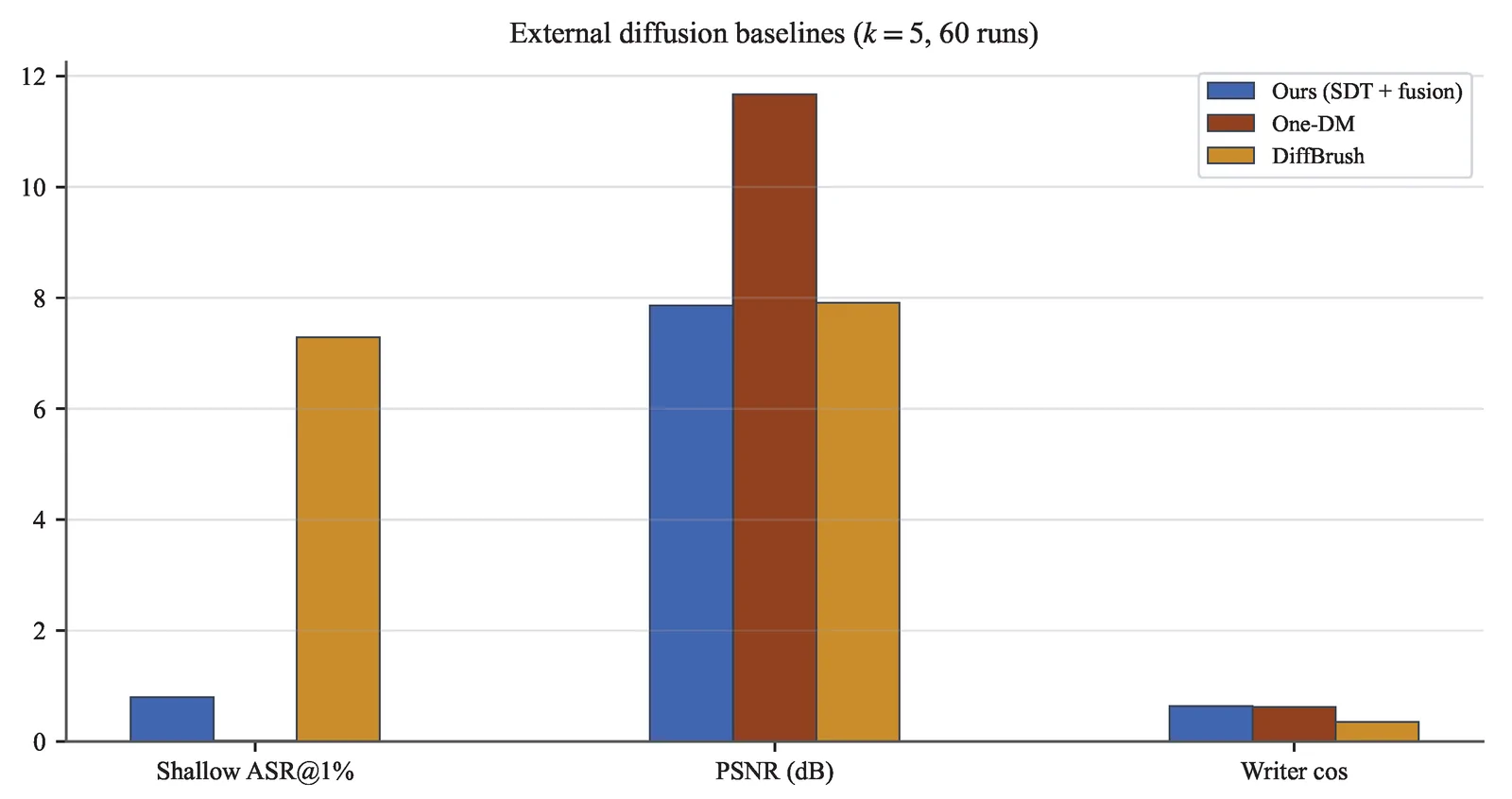
External diffusion baselines vs. trajectory-based dynamic fusion (k=5, 60 runs). The 12 held-out test writers are under the 73-writer writer-disjoint split.

To further investigate the relationship between stroke-width adaptation and verification system behavior, we conduct an exploratory security-oriented analysis framework. The threat model is a probing adversary who controls offline rendering parameters (writer-adapted widths) to test biometric verifier sensitivity under matched FAR operating points; verification pairs are constructed with writer-disjoint detector train/test splits. This analysis is intended as a diagnostic of detector sensitivity to width variations rather than an attack optimization study. We present the module as a controllable probing tool, not an attack-success booster. Figure 20 shows that rendered appearance (PSNR) remains comparable between generic and adapted-fused outputs. Table 9 and Figure 21 report ASR at three FAR levels for adapted-fused rendering. Table 10 complements this with a direct generic-fused vs. adapted-fused comparison on the same 60 runs and detectors, supporting the controllable-analysis claim: adaptation shifts detector responses measurably but not dramatically (Shallow CNN: 0.73% vs. 0.80% ASR at 1% FAR; ResNet-18: 3.97% vs. 3.77%; HOG+SVM: 9.78% vs. 9.55%). Shallow CNN is most sensitive overall; HOG+SVM is least sensitive. This detector-dependent pattern is consistent with controlled observations that verification outcomes vary substantially across backbone architectures under matched protocols [24]. Absolute ASR values remain modest under this protocol and should not be interpreted as operational spoofing success; the value lies in comparing rendering variants within a fixed verification pipeline. External diffusion ports (Table 11, Figure 22) reveal a fidelity and detectability trade-off: One-DM achieves lower Shallow ASR and higher PSNR, while our trajectory-based pipeline preserves the highest writer cosine and exposes width-level controllability absent in pixel generators. Because One-DM/DiffBrush are image-generation baselines rather than drop-in trajectory width estimators, the comparison is complementary under differing input and few-shot adaptation conditions (inputs, adaptation under our manifests, resolution, and comparable metrics).

### 4.9. Efficiency and Ablation Analysis

**Figure 23:**
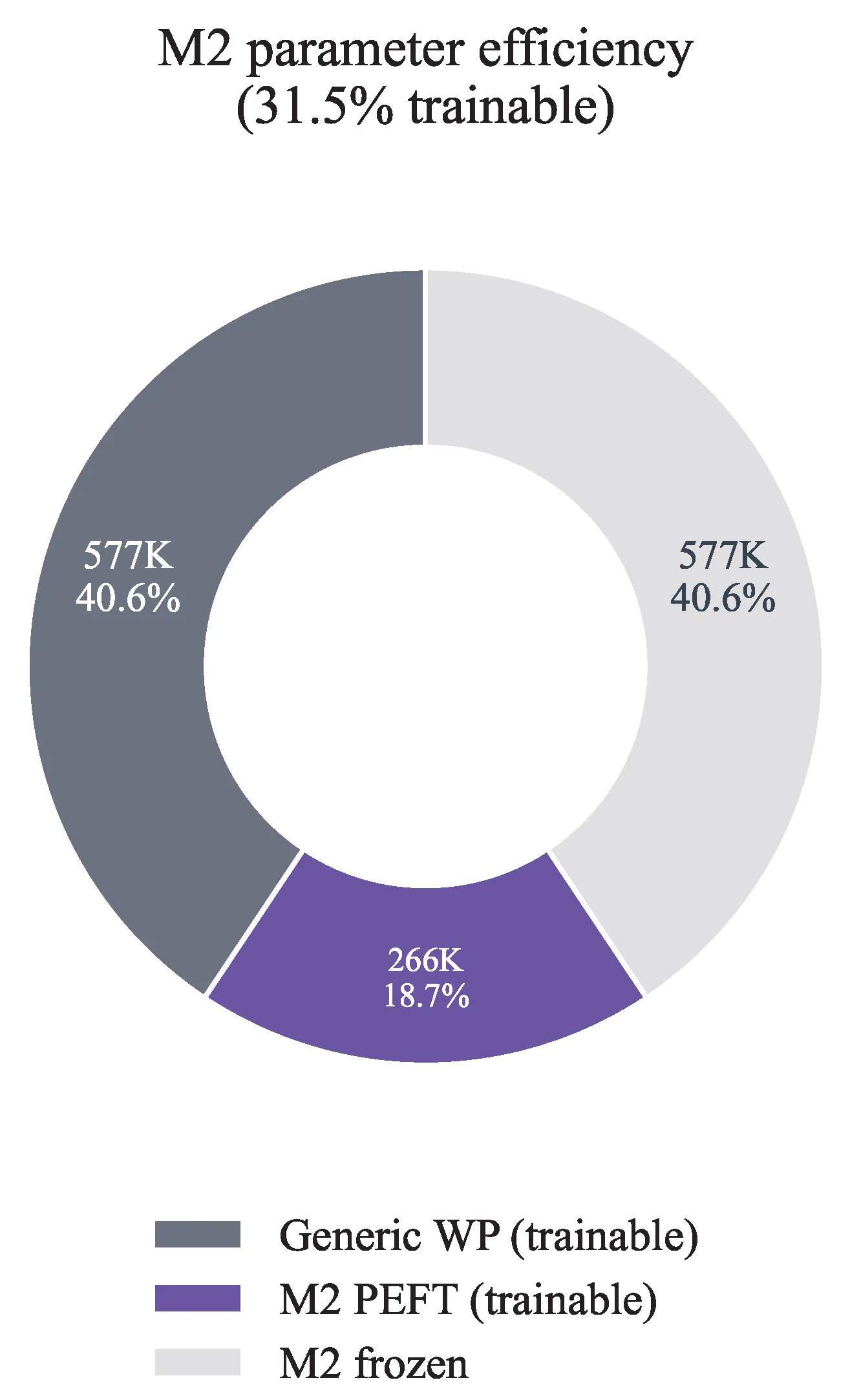
Parameter efficiency of writer-conditioned PEFT (slice percentages are rounded and may not sum exactly to 100%).

**Figure 24:**
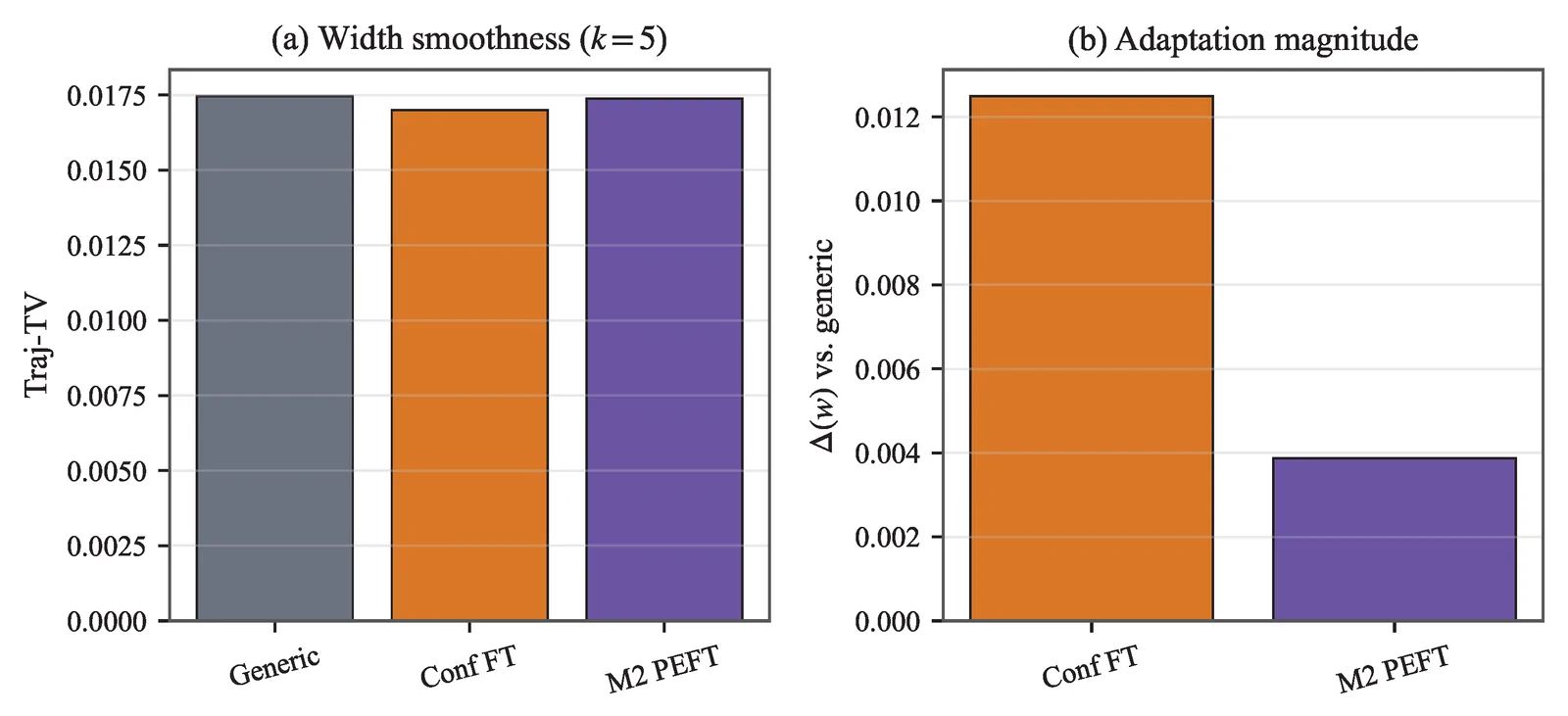
Adaptation magnitude: Conf FT changes widths more aggressively than PEFT.

**Figure 25:**
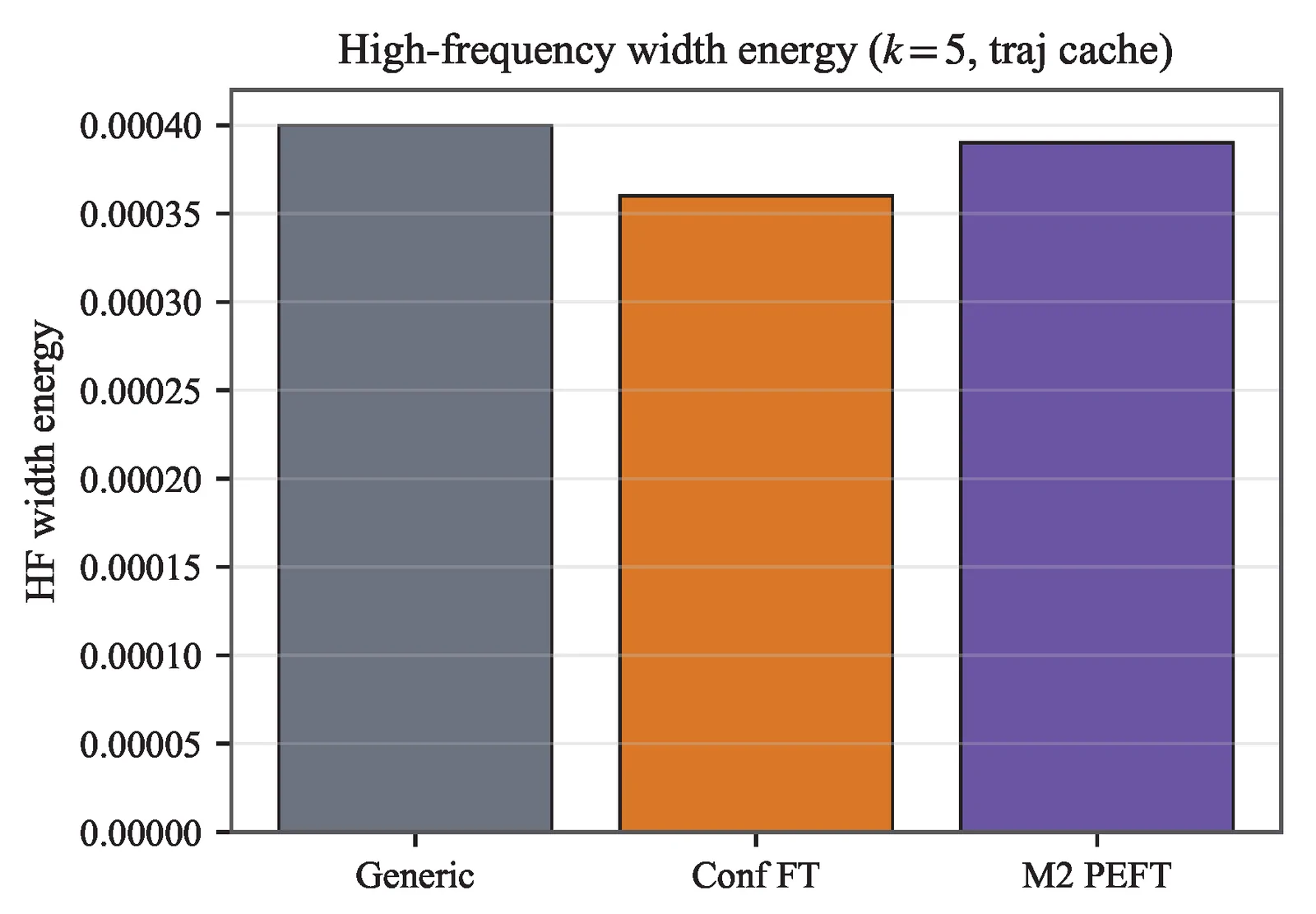
High-frequency width energy after adaptation.

**Figure 26:**
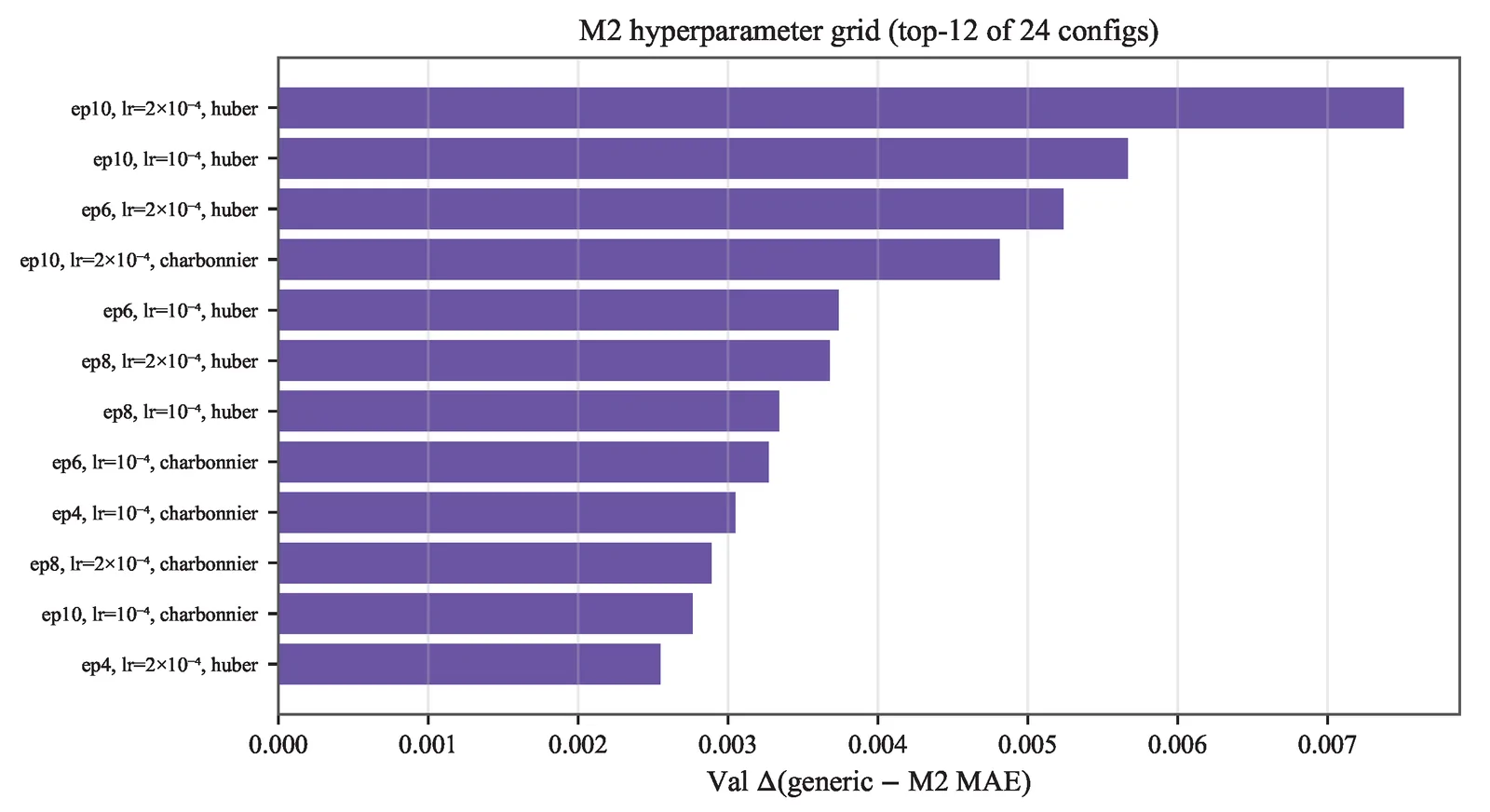
PEFT hyperparameter search on validation writers (top 12 of 24 configs).

**Figure 27:**
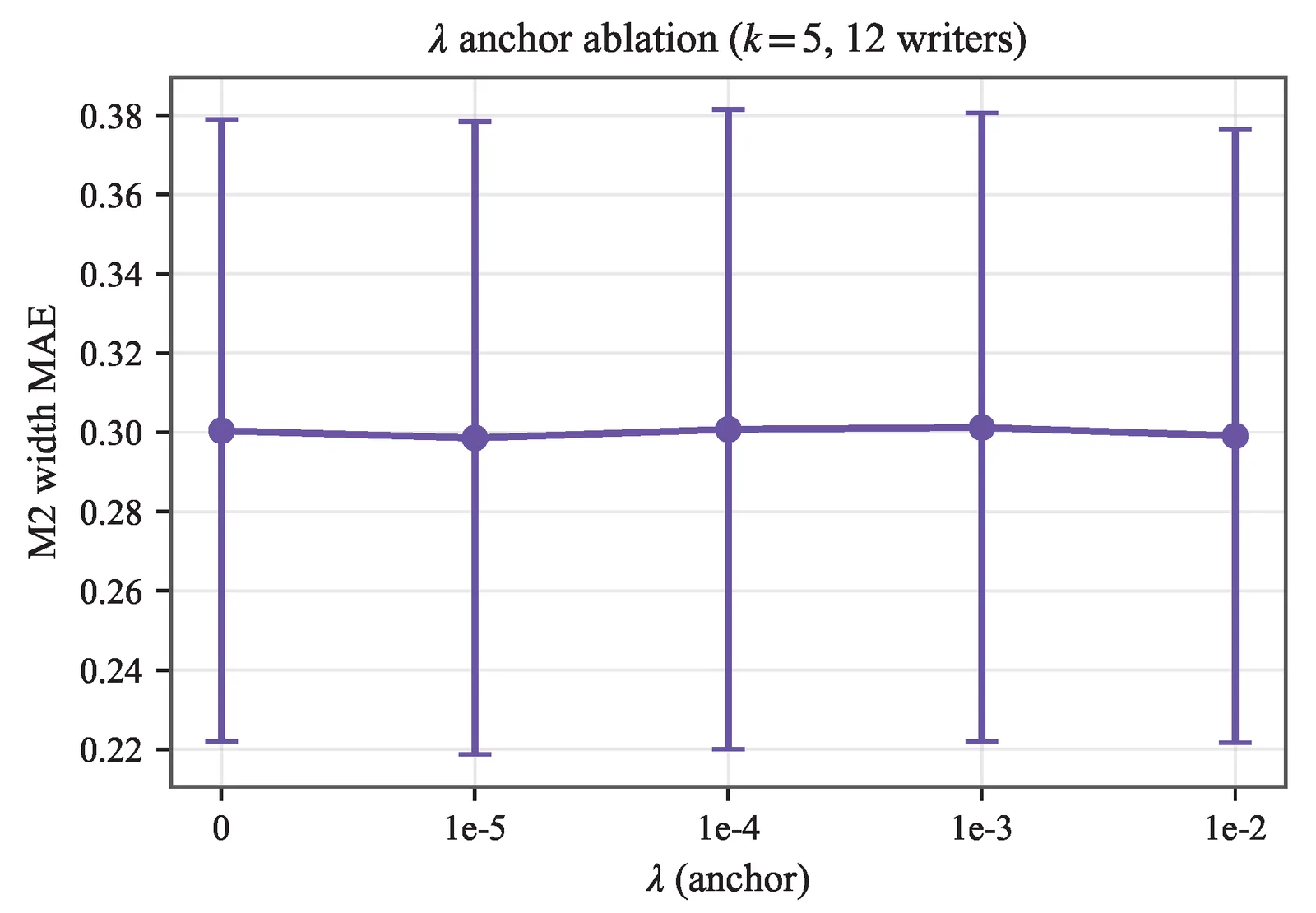
Anchor strength λ ablation for PEFT (k=5, 12 writers). The 12 held-out test writers are under the 73-writer writer-disjoint split.

Table 12 compares adaptation strategies. Compared with full fine-tuning, parameter-efficient adaptation significantly reduces optimization overhead (31.5% trainable parameters) while maintaining comparable inference latency (11.5 ms per 80-point trajectory). We additionally report forward latency ≈31.5 ms/sequence, WidthPredictor FLOPs$∼9.2\times 10^{7}$ (length 80), peak dummy-forward GPU memory ≈40 MB, and one adaptation epoch ≈5.4 s on a k=5 run; we do not over-claim “lightweight” beyond these measured numbers, and we do not market 31.5% trainable as extremely lightweight. Figure 23 summarizes the PEFT trainable-parameter split (rounded percentages). Traj-TV and HF energy (Figure 24 and Figure 25) show that PEFT edits widths more conservatively than Conf FT (Δ=0.004 vs. 0.013 vs. generic at k=5), indicating lower overfitting risk under limited writer samples rather than a separate accuracy claim. A 24-config validation grid did not improve test MAE over the default PEFT setting (Figure 26); the anchor-strength ablation is shown in Figure 27.

**Confidence and cue ablations.** Controlled ablations on the same queries support the operational confidence reading: random confidence yields run MAE 0.296 vs. dynamic 0.292 (beats dynamic on only 5/60; paired $p=1.0\times 10^{-9}$); inverse confidence yields 0.301 (5/60; $p=6.3\times 10^{-10}$); spatial β without confidence yields 0.296 (5/60 vs. dynamic); no rule prior (β=1) yields 0.303 vs. dynamic 0.292 (dynamic better 54/60; $p=2.7\times 10^{-9}$, $d_{z}\;\approx \;1.19$). Smoothing (kernel 1 vs. 3) has near-zero MAE effect and is presented as optional post-processing, not an accuracy contribution. Spatially varying coefficients alone therefore do not explain the gain.

**Style-preservation metrics.** On dynamic-fusion outputs, writer-level mean-width Pearson correlation is ≈0.73, between/within dispersion ≈2.06, and nearest-centroid writer classification is ≈26.7% vs. 8.3% chance. These are supportive but limited signals—not a standalone biometric identifier—and temper both memorization and over-strong writer-ID claims from width alone.

**Robustness vs. fixed fusion under matched perturbations.** Under matched blur/JPEG q=50/darkening, dynamic fusion is comparable under heavy blur (*p* n.s.), slightly better under JPEG (10/12 writers, p≈0.005) and darkening (9/12, p≈0.034), providing partial robustness evidence only.

## 5. Discussion

The experimental results reveal several important observations regarding few-shot writer-specific stroke-width adaptation. Unlike conventional prediction-oriented approaches, the proposed framework focuses on modeling the reliability variation among different stroke-width sources rather than simply optimizing average prediction accuracy.

### 5.1. Interpretation of Model-Only vs. Fusion-Stack MAE

Stage-A generic prediction attains strong model-only MAE (0.302; Appendix A) but does not include rule blending or the post-processing chain used at render time. Fixed-width support mean is lower still (0.285) by construction, yet yields near-zero temporal width variation and visually flat strokes (Figure 6a,b). This echoes a broader observation in visual synthesis that lower point-wise error need not imply better perceptual quality [31]. After validation tuning, dynamic fusion achieves 0.292 fused-pipeline MAE, improving over fixed fusion (0.303) and the generic Stage-A baseline, while retaining higher writer-level stability (Table 1). This resolves the apparent tension between model-only accuracy and deployed-stack performance: the deployed renderer intentionally blends learned widths with rule priors and applies post-processing for robustness, so its MAE should be evaluated against fixed fusion under the same pipeline, not against model-only outputs.

### 5.2. Why Fixed Fusion Is Insufficient

The comparison between fixed fusion and dynamic fusion further indicates that a constant fusion coefficient is insufficient for diverse writer conditions. Since different writers exhibit different stroke characteristics, assigning identical weights to all cases may lead to suboptimal adaptation (0.303 fused-pipeline MAE vs. 0.292 for dynamic fusion). Our confidence maps, cross-writer β distribution (Figure 11), confidence and error validation (Figure 13), and run-level stability analysis (Table 1) show that reliability varies spatially and across writers. The adaptive weighting mechanism provides a more flexible solution by considering writer-dependent reliability.

### 5.3. Role of Parameter-Efficient Adaptation

Regarding adaptation efficiency, parameter-efficient fine-tuning provides a practical compromise between personalization ability and optimization cost. Although full fine-tuning may achieve comparable or slightly different accuracy under certain settings, it requires substantially more trainable parameters and is more susceptible to overfitting when writer-specific samples are limited (Table 12, Figure 24 and Figure 25). PEFT is therefore treated as an efficient adaptation backend rather than a standalone scientific contribution.

### 5.4. Security-Oriented Implications

The security-oriented experiments should be interpreted as an *exploratory security-oriented analysis framework* rather than an attack optimization study or operational spoofing tool. Under the probing threat model with writer-disjoint detectors, ASR changes at 1% FAR remain small/inconsistent, so we frame the module as a controllable probing tool rather than an attack booster. The results demonstrate that synthesized handwriting may influence verification responses differently depending on the biometric detector characteristics (Table 9 and Table 10). Generic-fused and adapted-fused variants produce measurable but modest ASR shifts under the same protocol, which supports using the pipeline as a controllable probe of detector sensitivity rather than claiming spoofing superiority over external generators.

### 5.5. Positioning Relative to Diffusion Synthesizers

One-DM and DiffBrush achieve stronger pixel fidelity and, in some settings, lower verification ASR, but they do not expose controllable stroke-width reliability within a trajectory pipeline (Table 11). Comparisons remain complementary where input and few-shot adaptation conditions differ from our trajectory width estimator. This trade-off is consistent with broader discussions of diffusion synthesizers, which often excel at sample fidelity while controllability depends on how conditioning and editable intermediate representations are exposed [14,17,32]. Our approach is complementary: it improves a modular rendering stage that remains relevant when trajectories are cached or edited independently of pixels.

### 5.6. Limitations and Future Work

Despite the effectiveness demonstrated in our experiments, several limitations remain. First, the proposed framework mainly focuses on stroke-width adaptation and assumes that trajectory information is available from a fixed SDT generator; end-to-end co-training is not explored. Second, the primary evaluation uses 12 held-out test writers; the expanded HWDB1.1 check (28 test writers) covers seeds 0–1 only and is directional rather than a full five-seed claim; a complementary single-writer style-photo benchmark (my_style; Appendix C) confirms fusion-stack gains but cannot replace the writer-disjoint protocol. Third, the manual-click audit exposes circularity and absolute-scale mismatch and is not used as a fair fusion ranking; main conclusions do not depend on manual rankings. Fourth, learned gates are reported as an optional enhancement: they can lower HWDB pseudo-label MAE but require fusion-time training and do not transfer under a FontRNN generator swap. Fifth, pseudo-labels remain noisy (∼5 px mean deviation from an independent geometric reference on 15 audit images). Sixth, although the proposed method improves reliability-aware adaptation, it does not aim to replace large-scale generative models such as diffusion-based handwriting synthesis approaches. Future work will explore more comprehensive handwriting personalization by incorporating additional visual attributes and investigating larger-scale writer adaptation scenarios.

## 6. Conclusions

This study investigates the reliability imbalance problem in few-shot writer-specific stroke-width adaptation for trajectory-based handwriting synthesis. We find that different stroke-width sources, including learned predictions and handcrafted priors, exhibit varying reliability across writers, making fixed fusion strategies insufficient for personalized rendering.

Our confidence-guided dynamic fusion framework—with a deterministic confidence-to-β map as the primary operating point—adaptively blends learned and rule-based widths based on per-point confidence, where “reliability” denotes an operational blending prior. On a writer-disjoint protocol with 73 writers, it outperforms validation-selected fixed-β and model-only baselines in stability, worst-case error, and mean MAE (54/60 wins; 0.292 vs. 0.303). Manual audits and optional learned gates are reported as complementary evidence layers rather than replacements for the primary protocol. Security-oriented analyses further explore the relationship between width adaptation and verification system responses under multiple detectors, providing a controlled probing tool for system evaluation rather than claiming spoofing gains.

Future work will explore more comprehensive handwriting personalization by incorporating additional visual attributes and investigating larger-scale writer adaptation scenarios.

## Figures and Tables

**Table 1:** Primary fusion-stack evaluation at k=5 (60 runs): stability metrics on the deployed rendering stack. The 12 held-out test writers are under the 73-writer writer-disjoint split.

Method	Writer Std ↓	Worst-Writer ↓	Cross-Seed CV ↓	Win Rate ↑	Mean MAE
Fixed fusion	0.076	0.414	0.039	N/A	0.303
Dynamic fusion (ours)	**0.069**	**0.397**	**0.036**	**54/60**	**0.292**

Bold: best value in each column. ↓: lower is better; ↑: higher is better.

**Table 2:** Stratified fusion-stack comparison at k=5 by writer GT-TV (median split; tuned fusion config). The 12 held-out test writers are under the 73-writer writer-disjoint split.

Stratum	Runs	Fixed Fusion	Dynamic Fusion	Δ (Fixed − Dynamic)	Dynamic Wins
All writers	60	0.303	0.292	0.011	54/60
High GT-TV writers	30	0.339	0.323	0.017	30/30
Low GT-TV writers	30	0.266	0.261	0.006	24/30

**Table 3:** Confidence-subset fused-stack MAE at k=5 (pooled trajectory points, auxiliary; primary run-level means are in the primary fusion-stack tables). The 12 held-out test writers are under the 73-writer writer-disjoint split.

Subset	Fixed Fusion	Dynamic Fusion	Δ (Fixed − Dynamic)	Points
All points	0.448	0.371	0.077	1,228,233
Conf < median	0.379	0.365	0.014	614,116
Conf ≥ median	0.516	0.377	0.139	614,117
Conf ≥ 0.5	0.614	0.363	0.251	52,272

**Table 4:** Fused-pipeline width MAE after validation-only tuning ($\beta_{\min}=0.85$, $\beta_{\max}=0.99$, smooth kernel =3; light post-processing; 50 queries/run). The 12 held-out test writers are under the 73-writer writer-disjoint split.

*k*	Fixed Fused	Fixed-β	Dynamic-β	Δ
1	0.300	0.292	**0.289**	0.011
3	0.303	0.294	**0.291**	0.011
5	0.303	0.295	**0.292**	0.011
10	0.306	0.297	**0.294**	0.012

Bold: best value in each column.

**Table 5:** Run-level dynamic-β fused-pipeline MAE with 2.5/97.5% run percentiles (60 runs per *k*). The 12 held-out test writers are under the 73-writer writer-disjoint split.

*k*	Dynamic-β	2.5%	97.5%
1	0.289	0.172	0.407
5	0.292	0.172	0.399
10	0.294	0.174	0.399

**Table 6:** Manual stroke-width audit on 12 test writers (871 matched clicks): raw and LOWO-calibrated MAE (px). The 12 held-out test writers are under the 73-writer writer-disjoint split.

Method	Raw MAE (px)	Calibrated MAE (px)
Rule-only	10.21	4.73
Learned gates (rule-like under LOWO)	≈10.21–10.44	≈4.73
Dynamic (deterministic)	12.60	5.13
Fixed β=0.9	12.69	5.23
Model-only	12.97	5.51

**Table 7:** Learned-gate pseudo-label test MAE on the 12-writer protocol (multi-seed; Δ vs. dynamic). The 12 held-out test writers are under the 73-writer writer-disjoint split.

Method	Mean ± Std (3 Seeds)	Min	Δ vs. Dyn
Dynamic (deterministic)	0.292	—	—
Fixed β=0.9	0.295	—	+0.003
mlp_all	0.2637±0.0006	0.2631	−0.028
mlp_all_wide	0.2639±0.0005	0.2633	−0.028
mlp_conf	0.2765±0.0003	0.2763	−0.015
linear_conf	0.2781±0.00005	0.2781	−0.013
const	0.2905±0.00002	0.2905	−0.001

**Table 8:** Expanded HWDB1.1 split (180 writers; 125/27/28), k=5, seeds 0–1: directional check on 28 test writers (not a five-seed claim). Absolute MAE is not comparable to the 12-writer primary tables.

Seed	Dynamic	Fixed β=0.9	Fixed-Fused	Wins vs. Fixed-β	Wins vs. Fixed-Fused
0	0.476	0.483	0.482	24/28	23/28
1	0.483	0.488	0.488	27/28	23/28
Pooled	—	—	—	51/56	46/56

**Table 9:** Security-oriented analysis: ASR (%) at multiple FAR thresholds (k=5, adapted-fused, 60 runs). The 12 held-out test writers are under the 73-writer writer-disjoint split.

Detector	0.1% FAR	1% FAR	5% FAR
Shallow CNN	0.42	0.80	1.82
ResNet-18	0.63	3.77	13.93
HOG+SVM	6.27	9.55	22.77

**Table 10:** Generic-fused vs. adapted-fused ASR at 1% FAR (k=5, 60 runs): controllable rendering comparison within the same verification pipeline. The 12 held-out test writers are under the 73-writer writer-disjoint split.

Detector	Generic-Fused	Adapted-Fused	Δ (Adapt − Gen)
Shallow CNN	0.73	0.80	+0.07
ResNet-18	3.97	3.77	−0.20
HOG+SVM	9.78	9.55	−0.23

**Table 11:** External diffusion baselines (k=5, 60 runs; Shallow ASR at 1% FAR). The 12 held-out test writers are under the 73-writer writer-disjoint split.

Method	Shallow ASR@1%	PSNR (dB)	Writer Cos
Ours (dynamic fusion)	0.80	7.86	**0.638**
One-DM (Chinese port)	**0.02**	**11.67**	0.623
DiffBrush (Chinese port)	7.29	7.91	0.351

Bold: best value in each column.

**Table 12:** Adaptation efficiency profile (k=5, 80 trajectory points). The 12 held-out test writers are under the 73-writer writer-disjoint split.

Method	Trainable Params	Inference
Conf FT (full)	842,593 (100%)	11.5 ms
PEFT (adaptation backend)	265,824 (31.5%)	11.5 ms
Generic (no adaptation)	0	11.5 ms

Additional PEFT profile: forward ≈31.5 ms/sequence; WidthPredictor FLOPs $∼\;9.2\times 10^{7}$ (length 80); peak dummy-forward GPU memory ≈40 MB; one adaptation epoch ≈5.4 s on a k=5 run.

**Figure A1:**
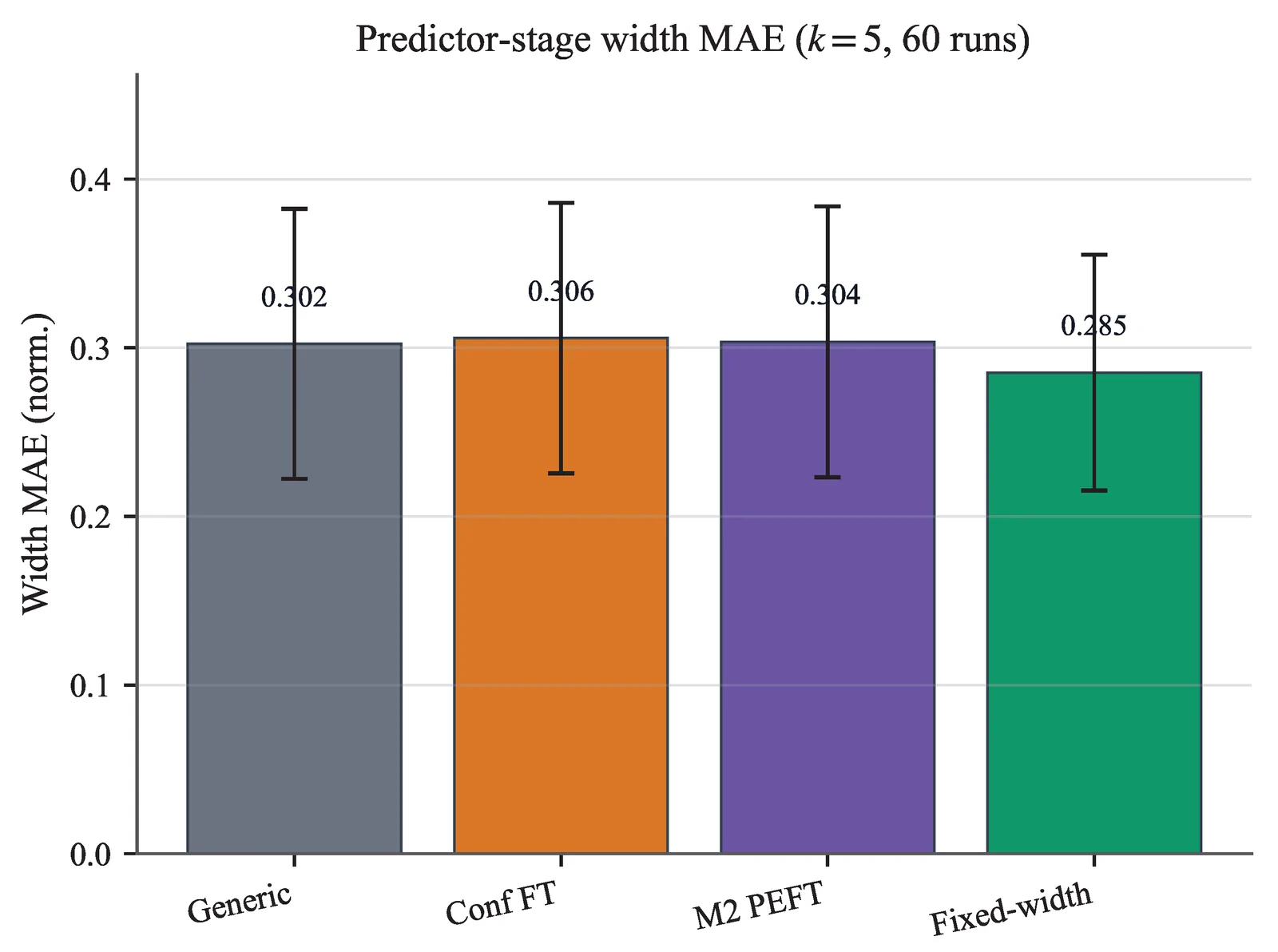
Auxiliary Stage-A predictor MAE at k=5 (60 runs). Not comparable to fusion-stack primary endpoints.

## Data Availability

Writer-disjoint split definitions, experiment scripts, evaluation logs, and figure-generation code supporting the reported results are included in the supplementary project materials bundled with this submission. HWDB character images are available from the CASIA online handwriting database (http://www.nlpr.ia.ac.cn/databases/handwriting/Home.html (accessed on 1 September 2025)). Cached SDT trajectories and trained width-predictor checkpoints used in the benchmark can be provided upon reasonable request to the author.

## Appendix A. Predictor-Stage-Baselines (Auxiliary)

Table A1 reports Stage-A predictor diagnostics at k=5 for transparency. Generic (0.302) and fixed-width support-mean (0.285) report model-only error without the deployed fusion-stack; dynamic fusion (0.292) evaluates the full renderer output $\beta_{t}\mu_{t}+\left(1-\beta_{t}\right)w_{t}^{\mathrm{rule}}$ after post-processing (Section 5.1).

**Table A1:** Auxiliary predictor-stage width MAE at k=5 (60 runs). The 12 held-out test writers are under the 73-writer writer-disjoint split.

Method	Mean MAE	Writer Std	Worst-Writer	Cross-Seed CV	Worst Run
Generic (no adaptation)	0.302	0.079	0.406	0.033	0.417
Fixed-width (support mean)	**0.285**	0.068	0.382	0.050	0.406

Figure A1 summarizes auxiliary Stage-A predictor MAE at k=5.

## Appendix B. Predictor-Stage MAE vs. Shot Count

Table A2 reports Stage-A predictor MAE for auxiliary comparison across adaptation backends and shot counts *k*. These numbers confirm that additional support primarily stabilizes rather than dramatically shifts average predictor error; they are not the primary evidence for the fusion contribution.

**Table A2:** Per-point width MAE on unseen-writers (mean±std over 60 runs per *k*; Stage-A only). The 12 held-out test writers are under the 73-writer writer-disjoint split.

*k*	Generic	Conf FT	PEFT	Fixed-Width
1	0.300±0.079	0.302±0.081	0.300±0.079	0.287±0.073
3	0.302±0.080	0.306±0.082	0.302±0.080	0.286±0.071
5	0.302±0.080	0.306±0.080	0.304±0.080	0.285±0.070
10	0.303±0.079	0.309±0.081	0.304±0.079	0.287±0.071

## Appendix C. Style-Reference Benchmark on my_style

To complement the writer-disjoint HWDB protocol, we evaluate width adaptation on the in-house my_style reference set (75 single-writer photos of one character class) under two complementary protocols. **Protocol A (direct-photos)** evaluates pseudo-width labels extracted directly from style-photos without SDT trajectory export. **Protocol B (SDT-pipeline)** exports 60 SDT-generated characters, adapts the predictor with k=5 style shots for 10 epochs ($\mathrm{lr}=5\times 10^{-5}$), and evaluates on 45 held-out rendered PNGs after validation-only fusion tuning ($\beta_{\min}=0.55$, $\beta_{\max}=0.94$, smooth kernel =13). Table A3 and Table A4 report point-wise MAE and temporal width variation (TV).

Both protocols show the same qualitative pattern: dynamic fusion substantially outperforms fixed fusion (+0.336 MAE on direct-photos; +0.275 MAE on SDT renders), while fixed-width scalar attains the lowest point MAE but zero TV. On the SDT-pipeline, adapted-only prediction slightly improves over generic (0.190 vs. 0.194 MAE), whereas direct-photo adaptation is noisier because pseudo-labels are extracted from photos without trajectory alignment. These results reinforce that point MAE alone favors constant-width baselines on homogeneous style-references, while dynamic fusion remains the preferred deployed-stack relative to fixed fusion.

**Table A3:** Protocol A: direct style-photo benchmark (57 test photos; k=0, 20 adapt epochs).

Method	MAE	Width TV
Generic model (no adapt)	0.183	0.017
Adapted model-only	0.221	0.010
Fixed-width scalar	0.091	0.000
Fixed fusion	0.569	0.006
Dynamic fusion (ours)	0.233	0.016

**Table A4:** Protocol B: SDT-pipeline benchmark (45 test PNGs; k=5, 10 adapt epochs).

Method	MAE	Width TV
Generic model (no adapt)	0.194	0.017
Adapted model-only	0.190	0.017
Fixed-width scalar	0.117	0.000
Fixed fusion	0.484	0.005
Dynamic fusion (ours)	0.209	0.009
